# Interlocking Interfaces for Enhanced Mechanical Properties in Bi-Component 3D Printing of Biodegradable Materials

**DOI:** 10.3390/mi17080937

**Published:** 2026-08-06

**Authors:** Maria Catana (Oancea), Catalin Tampu, Simona-Nicoleta Mazurchevici, Anastasios Tzotzis, Wojciech Sitek, Virgil Gabriel Teodor, Florin Susac, Monica Silvia Tatarciuc, Panagiotis Kyratsis, Ion Tiseanu, Cosmin Dobrea, Yujiao Ke, Viorel Păunoiu, Dumitru Nedelcu

**Affiliations:** 1 Department of Machine Manufacturing, Gheorghe Asachi Technical University of Iasi, Bd. Mangeron 59A, 700050 Iasi, Romaniasimona-nicoleta.mazurchevici@academic.tuiasi.ro (S.-N.M.); 2 Faculty of Engineering, Vasile Alecsandri University of Bacau, Calea Mărăşeşti 157, 600115 Bacău, Romania; 3 Department of Product and Systems Design Engineering, University of Western Macedonia, 50100 Kila Kozani, Greece; a.tzotzis@uowm.gr (A.T.); pkyratsis@uowm.gr (P.K.); 4 Faculty of Mechanical Engineering, Silesian University of Technology, ul. Akademicka 2A, 44-100 Gliwice, Poland; wojciech.sitek@polsl.pl; 5 Faculty of Engineering, Dunarea de Jos University of Galati, Street Domnească nr. 111, 800201 Galați, Romania; virgil.teodor@ugal.ro (V.G.T.); florin.susac@ugal.ro (F.S.);; 6 “Grigore T. Popa” Faculty of Dental Medicine, University of Medicine and Pharmacy Iasi, Universității Street 16, 700115 Iasi, Romania; monica.tatarciuc@umfiasi.ro; 7 National Institute of Laser Plasma and Radiation Physics, P.O. Box MG-36, 76900 Bucharest-Magurele, Romania; ion.tiseanu@inflpr.ro (I.T.); cosmin.dobrea@inflpr.ro (C.D.); 8 School of Mechanical Engineering, Yanshan University, Qinhuangdao 066004, China; 9 Technical Sciences Academy of Romania, Blvd. Dacia 26, 030167 Bucharest, Romania; 10 Academy of Romanian Scientists, Ilfov Street 3, Sector 5, 050044 Bucharest, Romania

**Keywords:** multi-material printing, PLA, PLA/PHA, biodegradable polymers, 3D printing interface, mechanical interlocking, mechanical properties, fracture behavior, X-ray computed tomography (XCT), structural integrity, polymer composites, interfacial adhesion, defect analysis

## Abstract

Additive manufacturing has evolved beyond monomaterial fabrication, enabling the integration of dissimilar polymers within a single structure to achieve spatially tailored properties. In Fused Filament Fabrication (FFF), however, the discrete, layer-wise deposition and inherent material incompatibilities make the interfacial region a critical determinant of structural integrity. Rather than acting as a simple boundary, the interface governs stress transfer, damage initiation, and failure propagation, especially in biodegradable polymer systems where thermal and rheological mismatches are pronounced. This study investigates bi-component FFF structures manufactured from PLA and PLA/PHA using mechanically interlocked interface geometries (T-type and dovetail configurations). Mechanical performance was assessed through tensile, flexural, and Charpy impact testing, complemented by fracture analysis, surface topography evaluation, and X-ray Computed Tomography (XCT) for internal defect characterization. The results establish correlations between interface design, defect distribution, and overall structural response.

## 1. Introduction

Over the past two decades, additive manufacturing (AM) has undergone significant advancements. Additive manufacturing, originally for quick prototyping, today makes sophisticated, customizable functioning components [1,2,3,4]. FFF is popular in industry and academia. Due to its cost-effectiveness, equipment simplicity, and compatibility with many thermoplastic polymers [1,2,5]. Layer-by-layer molten filament deposition provides internal structures and a laminated microstructure, with extruded route fusing determining mechanical properties [5,6,7].

Fused Filament Fabrication (FFF) multi-material or bi-component printing combines two or more materials into one component using multiple extruders or sequential filament feeding [8,9,10]. Thus, structures can have adaptive responses, localized functionalization, flexible and rigid zones, and gradient stiffness [8,11,12,13]. Poor interlayer adhesion, processing susceptibility, and localized porosity are FFF problems. Multi-material components’ structural stability depends on contact [8,11,14,15,16,17].

Polymer rheological and thermal compatibility, intermolecular diffusion, wetting, and interface design—from planar contacts to mechanically interlocked topologies—impact interface quality. Cracks spread with weak contact, especially in small places. Tensile, shear, and impact strength decrease [5,11,14,18]. Synthesis studies show that interface management is the biggest technological hurdle to large-scale industrialization of multi-material additive manufacturing components [8,9,11,12,13,19].

These advances have raised interest in biodegradable polymers and biocomposites. A popular biopolymer in FFF is polylactic acid (PLA), which is renewable, biodegradable, and efficient at low temperatures [2,6,20,21,22,23]. PLA has good mechanical properties; however, Hussain et al. suggest changes for biological uses [20]. After examining PLA, Rajendran, Kurowiak, Adekoya et al. showed that processing and additives balance biocompatibility, toughness, and regulated degradation [24,25,26,27].

Fused Filament Fabrication (FFF) polymeric materials were studied by Fico et al. PLA-based biocomposites with natural particles or fibers can match or exceed standard polymers in mechanical qualities while being environmentally benign. PLA biocomposites and biodegradable FDM/FFF materials are studied by Samykano, Sandanamsamy, and Shunmugasundaram. The arming phase, filament extrusion, and printing parameter ratios are crucial [6,7,28,29,30]. Palm fibers, continuous plant fibers, and metallic particles help PLA biocomposites stiffen and strengthen. They introduce printing repeatability and consistency difficulties [7,29,31,32,33,34].

Recently, PLA has been explored with PHA, PBS, PBAT, and other biodegradable composites. Olonisakin et al. found that PLA/PHA, PLA/PBS, and ternary combinations improve stiffness, toughness, and processability. Apply to gradient structures or sections with distinct characteristics [28]. A PLA/PHA hybrid material rheological investigation by Torabi et al. indicated that hot elongation and flow properties are critical for printing stability and internal fault prevention [35]. Vidakis et al. examined PHA mechanical properties and printing circumstances, finding biopolymer optimization inadequate [36]. Improvements may require tight FFF process regulation to extend applicability. Recent PLA nanocomposites research using nanoparticles, graphene, and metals supports this [31,33,34].

Dey et al. reviewed FFF filament-type material synthesis. Classic thermoplastics, bioplastics, and biocomposites were used. Materials, printing settings, and deposition techniques affected mechanical properties [1]. Layer thickness, extrusion temperature, and part orientation affected FFF PLA components, according to Cojocaru et al. [5]. Process windows limit property variability in industrial applications. Several studies associate FFF characteristics with biopolymers like PLA-lignin and other biocomposites’ tribological or dynamic-mechanical capabilities. Studies show that stratified microstructure and fault density drive performance [2,30,37,38].

Recently, the multi-material printing literature has grown. Nazir et al. examined MMAM (multi-material additive manufacturing) concepts, applications, and issues. Many material combinations struggle with interactions despite equipment and design breakthroughs [8]. Verme et al. added “multiplicity” to MMAM to combine materials and processes [11]. Voxel-level modeling and control were emphasized [11]. Nipu et al. prioritize multi-material interface design research [12].

The first synthesized multi-material additive manufacturing was in 2016. Wang et al. showed that accurate filament diameter switching and steady flow rates are necessary for surface replication in multi-material FDM [9,39]. Ghasemi et al. studied design optimization and process parameter control in MMAM, particularly in architectural and construction contexts, but their conclusions apply to all multimaterial polymer structures [13]. Yu et al.’s computer multi-material design method relates material distribution to strength and deformability [19].

The research uses fluoropolymerization-deposited polymeric material interactions. Ribeiro, Carneiro, and Ferreira da Silva examined PLA–PLA and PLA–TPU multi-material specimens with planar, U, T, and dovetail contact geometries. T-type and dovetail joints boost mechanical strength regardless of material compatibility [14]. Similarly, Dairabayeva et al. studied face-to-face, U, T, dovetail, encapsulation, and overlap geometries in single- and multi-material combinations. Macroscopic connections boost tensile strength and modify failure mode [40].

A study by Kluczyński et al. [18] demonstrated that joint arrangement and deposition technique affect adhesion between modified PLA and ABS, with interlocking portions rerouting crack course within the material rather than just along the interface. Altuntaş, Coker, and Yavaş observed that biomimetic “suture-type” interfaces between PLA (rigid) and TPU (flexible) create more robust structures with volumetric interlocking regions dictating failure [6,15,19,41].

Recent studies emphasize non-geometric methods for interface strengthening. To create interlaced infills in multi-material zones, Mustafa et al. sliced materials using layered depth pictures. Superimposed and volumetrically connected materials reduce interface resistance [21]. Sorimpuk et al. investigated PLA/TPU thermoformability for rigid-flexible composites. Stable joints with proper form and strength recovery can occur at particular temperatures [42]. Surface treatments, chemical activation, and annealing have been examined for FFF PLA–TPU adhesion. These studies indicated that printing conditions and local interfacial treatments affect bond quality [43,44,45].

These works use PLA, TPU, or ABS without biodegradable polymers in selected geometric designs. Fused Filament Fabrication (FFF) with biodegradable filaments has shown promise for sustainable structures [2,6,7,29,30,33,36,46], but multimaterial topologies with complicated interactions lack experimental evidence. FDM-printed PLA-based polymers’ tribological and dynamic-mechanical properties are sensitive to processing settings and internal architecture, according to Nedelcu et al. Multiple interfaces make this more crucial [37].

Integrating biodegradable materials, mechanical interlocking interfaces (simple T, in the mirror, turned around 2T, simple dovetail 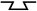, in the mirror, turned around 2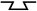), and FFF process parameters is poorly studied. Thus, design techniques and experimental correlations cannot transform biodegradable multimaterial components from sensitive interfaces to durable structural sections. This study examines samples from UltraFuse PLA Blue and PLA/PHA Shining Silver bicomponent 3D printed with the above interfaces, which undergo tensile strength, bending, Charpy impact, fracture, topography, and X-ray Computed Tomography (XCT) tests to evaluate mechanical properties, surface topography, and void analysis.

## 2. Materials and Methods

The materials used in the experimental research are UltraFuse PLA Blue and PLA/PHA Shining Silver. UltraFuse PLA Blue is one of the most widely used materials in 3D printing, ensuring high-quality prints. Advantages of choosing UltraFuse PLA [2]: wide range of colors; easy to print; repeatability; superior properties; relatively low printing temperatures; and low shrinkage. PLA/PHA Shining Silver, developed by Colorfabb, is a PLA-based filament enhanced with PHA (polyhydroxyalkanoate), which makes the filament tougher, with good layer adhesion and reduced warping tendencies [5]. The tensile tests were conducted in accordance with ISO 527, using the Ultimaker 3 Extended 3D printer (UltiMaker, Utrecht, Netherlands), equipped with two print heads, available at the Faculty of Engineering of the “Dunărea de Jos” University of Galați. The printing parameters were as follows: 100% infill, flat orientation of the specimens, build plate temperature of 60 °C, print speed of 60 mm/s, and extruder temperature of 215 °C (Table 1).

The 3D printing of the tensile test specimens was carried out according to the experimental plan (Table 2), in which the input parameters were the deposited layer thickness, the shell number, and the interface geometry. Each input parameter had two levels: interface geometry (T and 2T in the mirror, turned around), with the same interface length, number of shells (2 and 4), and layer thickness (0.1 and 0.3 mm). For the dovetail interface, the same experimental setup was used; the only parameter changed was the interface type (dovetail and in the mirror, turned around). In the case of interfaces at +1 level, we used the notations 2T and 2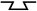. Three samples were printed for each experiment.

The interface types with dimensions are presented in Table 3. The length of the interfaces is constant (26 mm) regardless of their type. Before printing, both filaments were dried at a temperature of 45 C for 4 h using the BOV-V45F drying oven.

The uniaxial tensile tests were conducted at the “Gheorghe Asachi” Technical University of Iași, Faculty of Materials Science and Engineering, using an Istron 3382 universal testing machine (Instron, Norwood, MA, USA), at a constant speed of 1 mm/min in accordance with ISO 527-3:2003 [47], the distance between the grips was 115 mm, the data acquisition rate was 10 Hz, and the tests were conducted at room temperature (23 °C).

The impact strength of test specimens printed from UltraFuse PLA Blue and PLA/PHA Shining Silver materials was determined using the Charpy method in accordance with the SR EN ISO 179-1 standard [48]. The Charpy impact test was performed using the ZwickRoell HIT25P pendulum (ZwickRoell GmbH & Co.KG, Ulm, Germany), which is designed for testing plastics; it has a nominal energy of 0.209 J, without friction correction, and the pendulum was positioned at an angle of 104.22°. The test parameters used to determine impact resistance were a velocity of 2.9 m/s and a 5 J pendulum.

The bending tests were conducted at the “Gheorghe Asachi” Technical University of Iași, Faculty of Mechanics, using an Istron 3382 universal testing machine with a load of 1 kN and a speed of 2 mm/min.

The bending strength was determined using the three-point method, with the test specimens placed on two supports at a distance of L = 60 mm; a vertical force was then gradually applied to the center of the specimen, exactly halfway between the supports. Thus, for each test specimen, the applied force (F) and the vertical displacement (deflection) were recorded. The specimens had the following dimensions: test specimen length 80 mm, thickness h = 4 mm, and width b = 10 mm.

**Figure 1:**
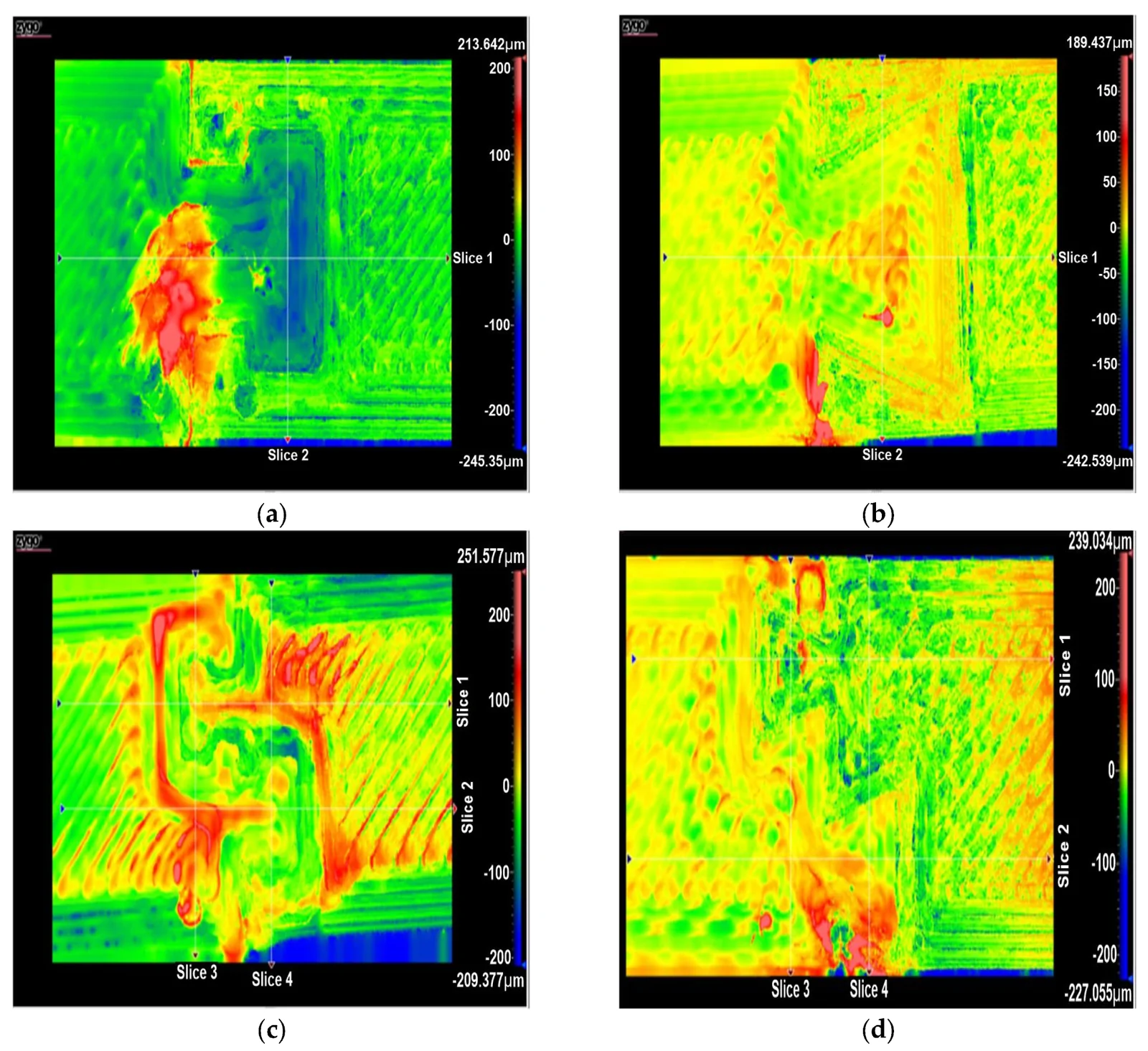
Topographic analysis: slice 1, slice 2: (**a**) T interface, (**b**) 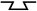 interface; slice 3, slice 4: (**c**) 2T interface, (**d**) 2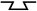 interface.

The topography was measured using the Zygo Zegage optical interferometric profilometer (ZYGO Corporation, Middlefield, CT, USA), a non-contact instrument designed for high-resolution 3D topography measurement. The analysis was performed in two directions (slice 1—longitudinal, slice 2—transverse) for single interfaces, and in the case of inverted (double) interfaces, two longitudinal directions (slice 1, slice 3) and two transverse directions (slice 3, slice 4) were analyzed as shown in Figure 1.

X-ray Computed Tomography (XCT): Non-destructive 3D imaging was performed using a microfocus high-energy X-ray generator operating at a maximum voltage of 225 kV and a current of 2 mA. The system utilized a tungsten anode to produce the X-ray beam. The transmitted radiation was captured by a high-resolution flat panel detector featuring a 2048 × 2048 pixel matrix with a 200 μm pixel size and a 16-bit dynamic range. To ensure optimal image quality and spatial accuracy, the geometric magnification was adjusted to achieve a voxel resolution of 40 μm for all investigated samples.

Experimental control and data acquisition were managed through dedicated software applications. The raw projection data were processed using advanced CT reconstruction software to generate 3D volumetric datasets. These volumes were then imported into VGStudio Max, a specialized 3D tomographic visualization and metrology software, for further analysis.

## 3. Results and Discussion

### 3.1. Tensile Tests

The tensile tests performed on the multi-material samples manufactured by FFF highlighted the decisive role of the interface design and the processing parameters on the mechanical behavior. For all four analyzed configurations (T, in the mirror turned around (2T), dovetail (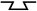), and in the mirror turned around (2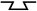)), the failure mode and the achieved strength level were strongly correlated with the interconnection mechanism between the two materials and with the filament orientation relative to the loading direction.

The maximum mean results of the uniaxial tensile tests on the multi-material samples printed from UltraFuse PLA Blue–PLA/PHA Shining Silver for the four interface models are presented in Table 4.

**Figure 2:**
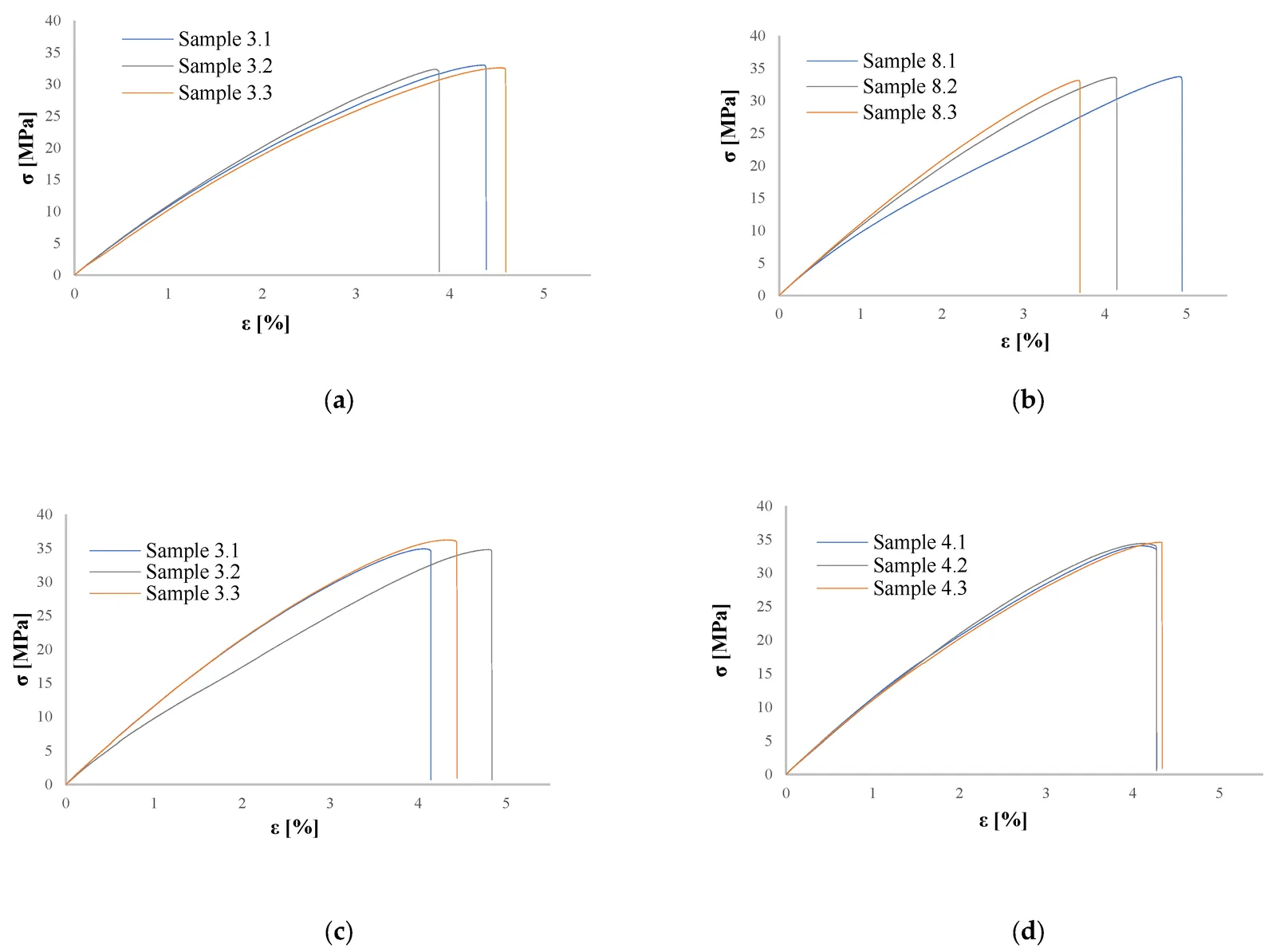
Stress–strain behavior of bicomponent UltraFuse PLA Blue–PLA/PHA Shining Silver 3D-printed samples: (**a**) T; (**b**) 2T; (**c**) 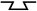; (**d**) 2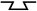.

Figure 2 shows the stress–strain behavior of samples with T, 2T, 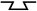, and 2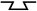 interfaces, printed from UltraFuse PLA Blue and PLA/PHA Shining Silver materials.

**Figure 3:**
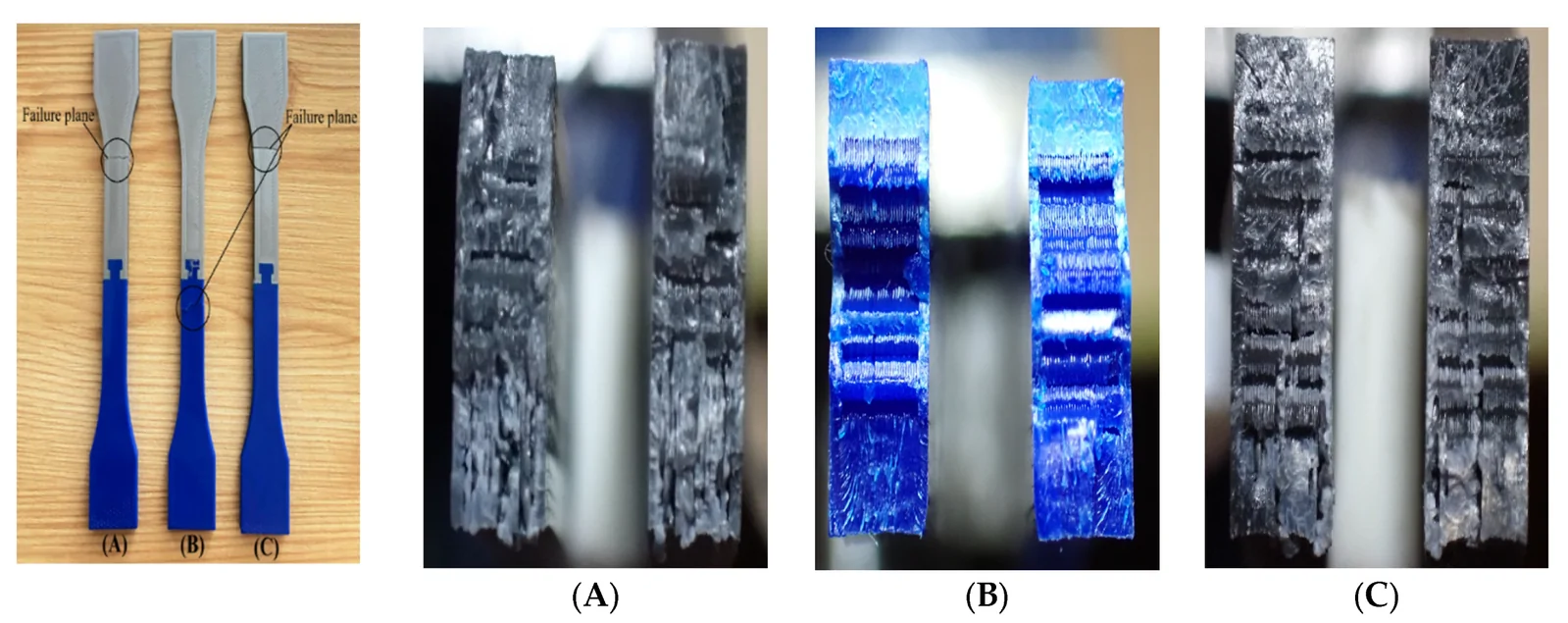
Fracture regions of the T-interface samples: cross-sectional view of failure sample (**A**–**C**), left images—area above the break; right images—area below the break.

Experiment 3 (T interface): In Experiment No. 3 (Figure 3A,C), samples 3A and 3C fractured at a relatively large distance from the interface; the material that failed was PLA/PHA Shining Silver, which was outside the fracture zone recommended by ISO. Sample 3B can be seen to have failed in the UltraFuse PLA Blue material zone, with a maximum tensile strength of 32.36 MPa (Table 5). All three samples exhibited a higher tensile strength, closer to the value specified by the manufacturer (34.7 MPa), due to the four shells used during the 3D printing.

**Figure 4:**
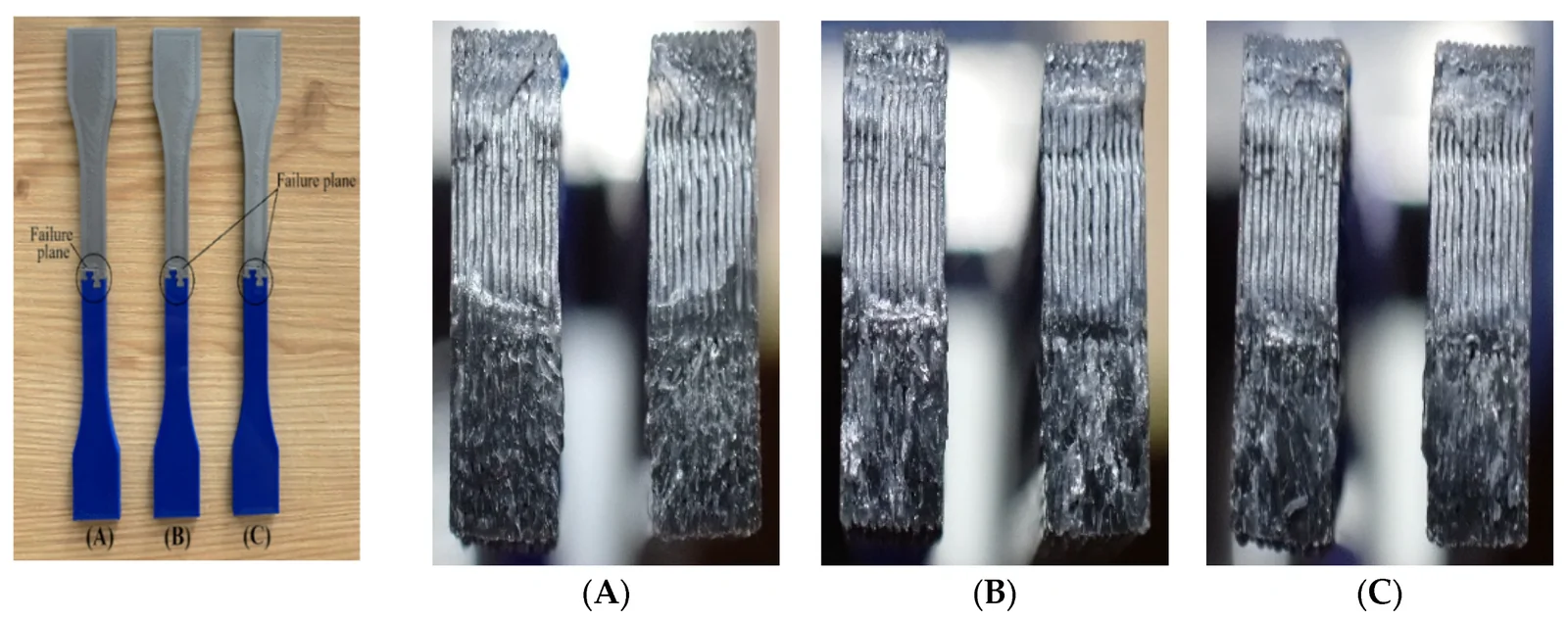
Fracture regions of the 2T-interface samples: cross-sectional view of failure sample (**A**–**C**), left images—area above the break; right images—area below the break.

Experiment No. 8 (2T interface): The fracture occurred in the immediate vicinity of the interfaces in the Shining Silver PLA/PHA material (Figure 4). It appears that the adhesion between the deposited shells is weaker in this material than in UltraFuse PLA, such that the layers detached in the T-head area of the PLA/PHA Shining Silver material, after which the break occurred in this weakened area.

**Figure 5:**
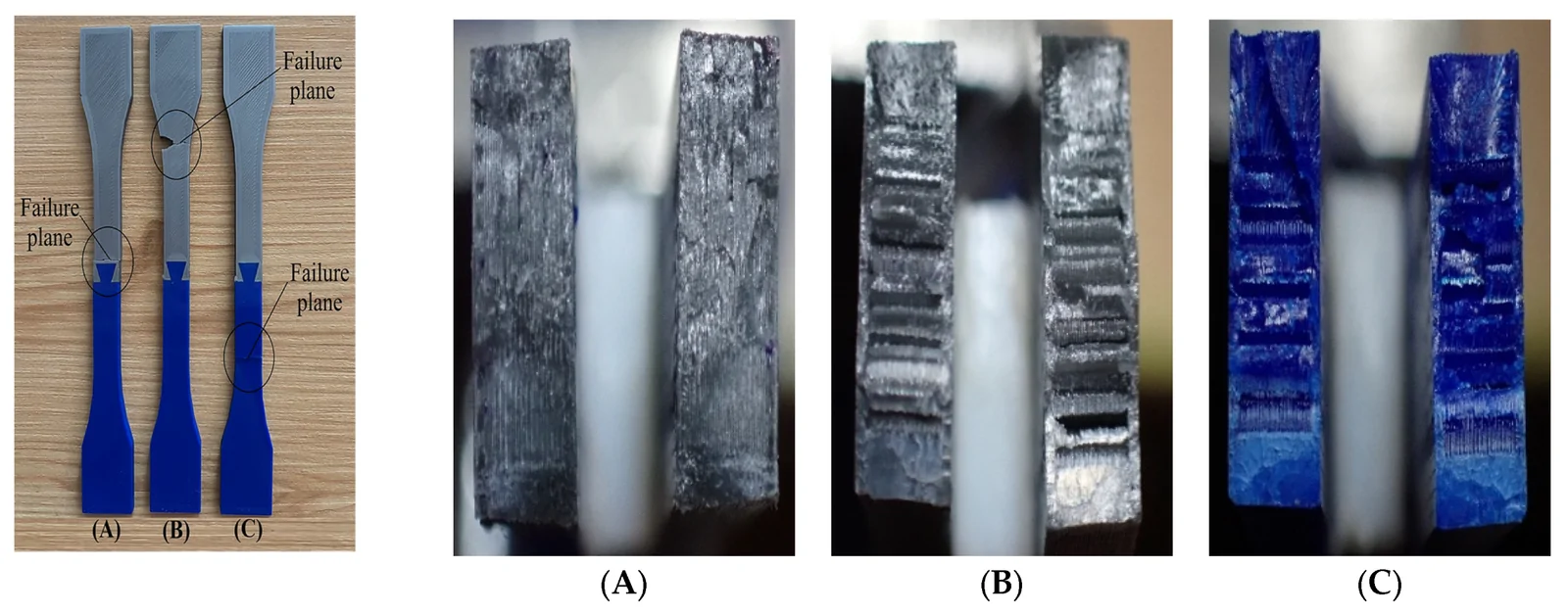
Fracture regions of the 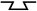 interface samples: cross-sectional view of failure sample (**A**–**C**).

Experiment 3 (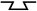 interface): The results shown in Figure 2c,d revealed the most varied fracture patterns of the entire experiment. The fracture in the first A sample occurred in the PLA/PHA Shining Silver material in the area where the shells are oriented transversely to the direction of the tensile force (where adhesion is minimal). In sample B, the fracture occurred in the PLA/PHA Shining Silver material. Due to the four shells (as explained in the T interface), the mechanical properties are higher than those recorded in the first two experiments. The fracture micrographs in Figure 5 confirm that all samples with a dovetail interface fractured in areas near the interface, particularly in the PLA/PHA Shining Silver material, where the transverse shells create mechanical discontinuities.

**Figure 6:**
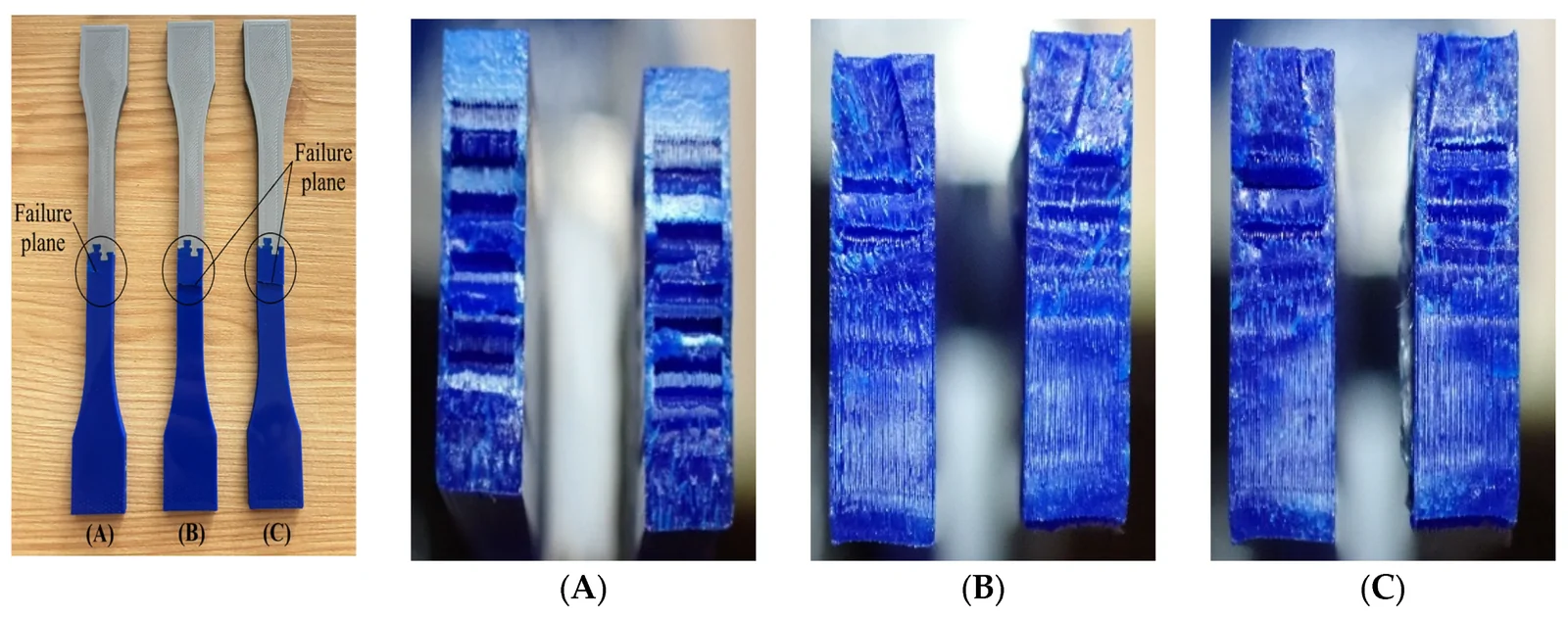
Fracture regions of the 2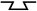 interface samples: cross-sectional view of failure samples (**A**–**C**).

Experiment 4 (2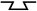 interface): In the first specimen (A) (Figure 6), the fracture occurred approximately 10 mm from the interface. In the other two specimens (B, C), the fracture was observed near the interface, in the UltraFuse PLA Blue region, where the shells are oriented transversely to the direction of the tensile force. The mechanical property results are the best and most homogeneous (minimal dispersion) across the entire experimental setup.

Sample failure during tensile testing generally had two causes:

– Failure in the section consisting of shells oriented in the direction of the tensile force and the infill;
– Delamination between the shells in the sections where the shells were oriented perpendicular to the direction of the tensile force.

Increasing the number of shells had a positive effect because their orientation along the direction of the tensile force resulted in improved strength.

The 2 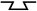 interface allowed for a more uniform distribution of stresses in the interface region (resulting in lower stresses), which led to improved mechanical properties at fracture.

To provide a complete picture of the tensile test results across the entire experimental range, Table 5 and Table 6 present the results obtained for all eight experiments.

### 3.2. Bending

**Figure 7:**
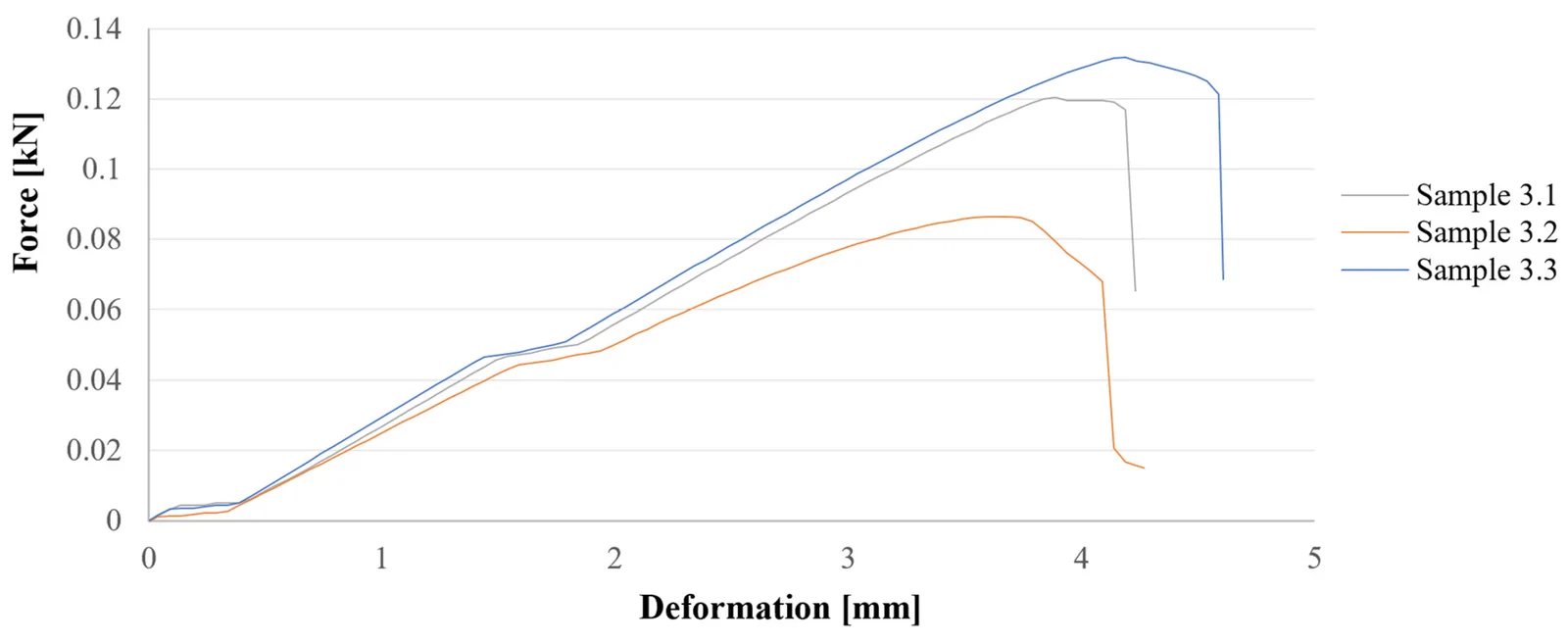
Force–displacement curves for Experiment 3: T interface.

**Figure 8:**
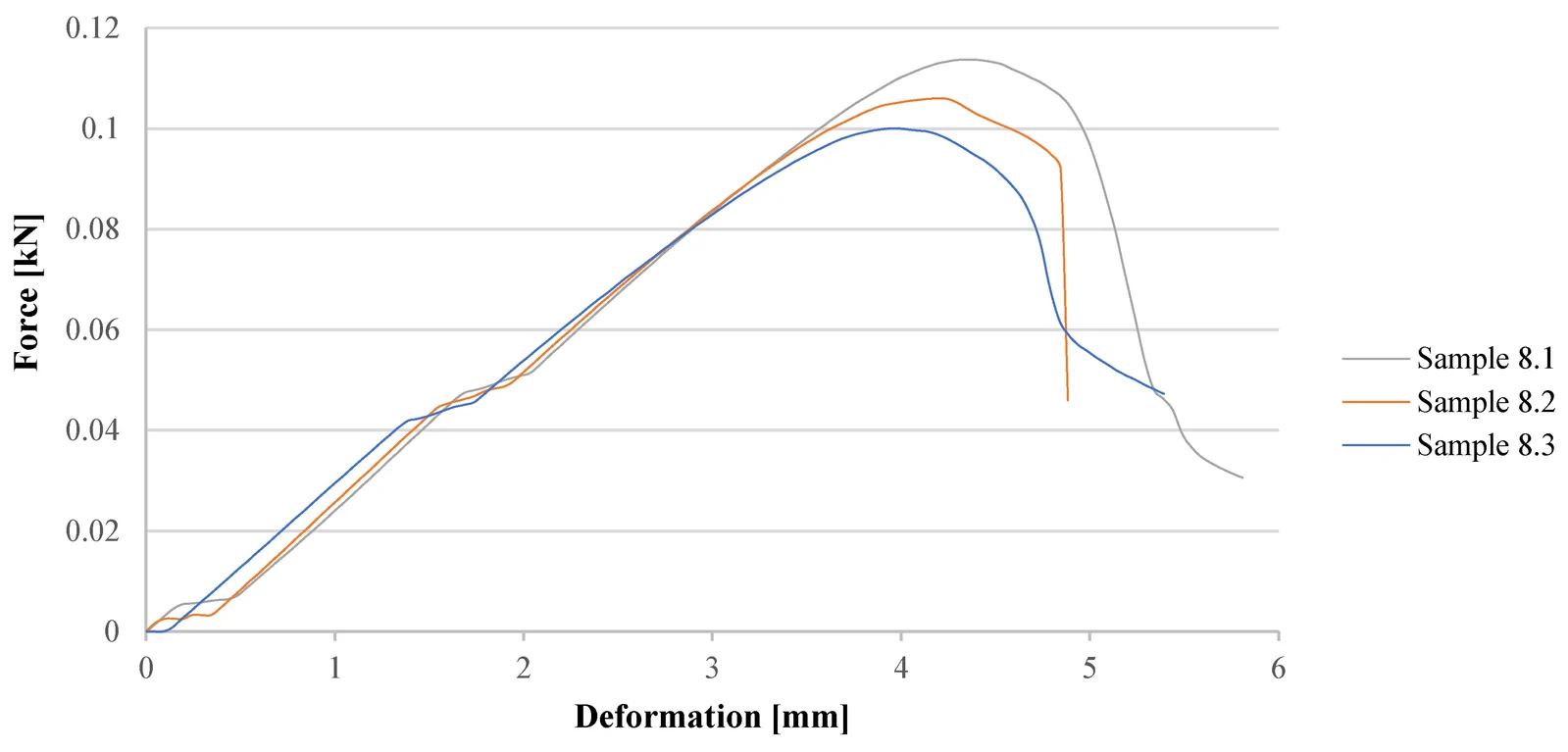
Force–displacement curves for Experiment 8: 2T interface.

The force–displacement curves for the two experiments (T interface and 2T interface) printed using UltraFuse PLA Blue and PLA/PHA Shining Silver materials are shown below (Figure 7 and Figure 8).

Figure 7 shows that sample 3.3 performed best, with a bending strength of 59.75 MPa (Table 7); it withstood the highest load before failure (F = 0.13 kN) and exhibited the greatest deflection (4.61 mm). In conclusion, sample 3.3 has the best mechanical bending strength.

**Figure 9:**
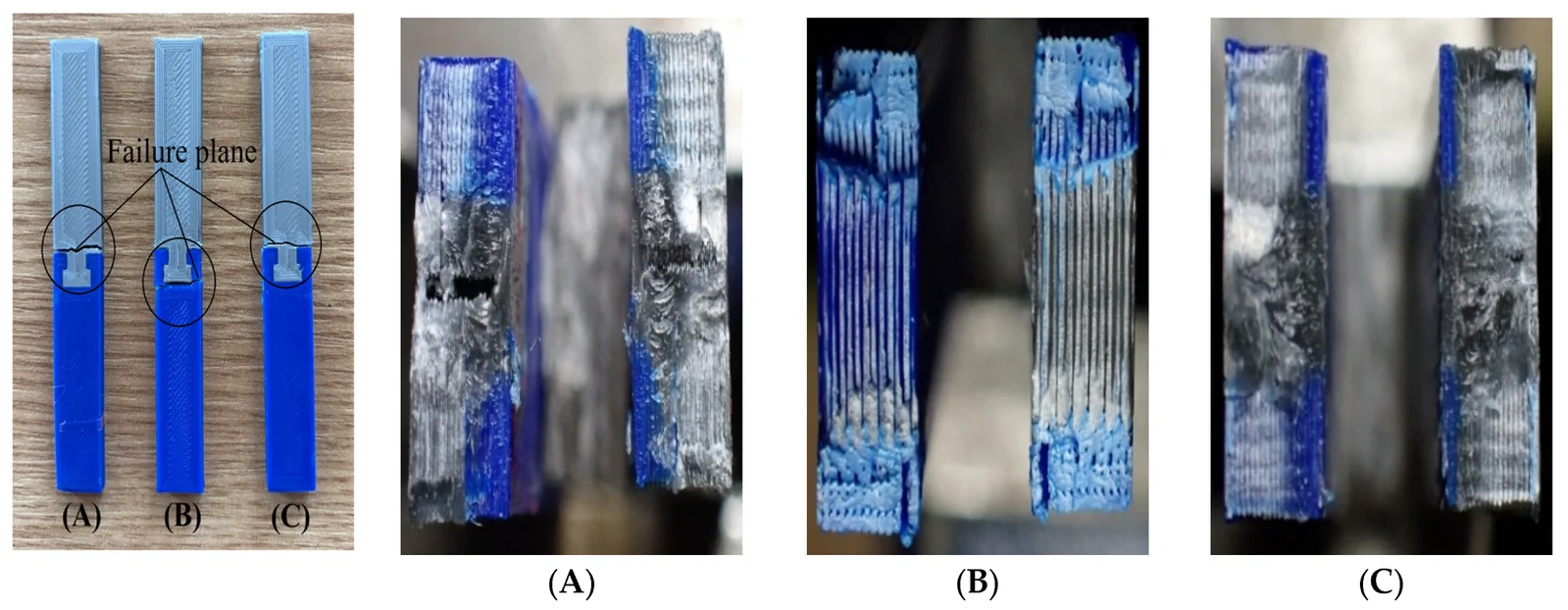
Fracture zones of sample 1, with T interface: (**A**) cross-sectional view of the fracture zone of sample 3.1; (**B**) cross-sectional view of the fracture zone of sample 3.2; (**C**) cross-sectional view of the fracture zone of sample 3.3.

Figure 8 shows that sample 8.1 performed best, with a bending strength of 51.52 MPa (Table 7); it withstood the highest load before failure (F = 0.11 kN) and exhibited the greatest deflection (5.81 mm). In conclusion, sample 8.1 has the best mechanical bending strength. Figure 9 shows the fracture zones for Experiment 3, with a T interface.

**Figure 10:**
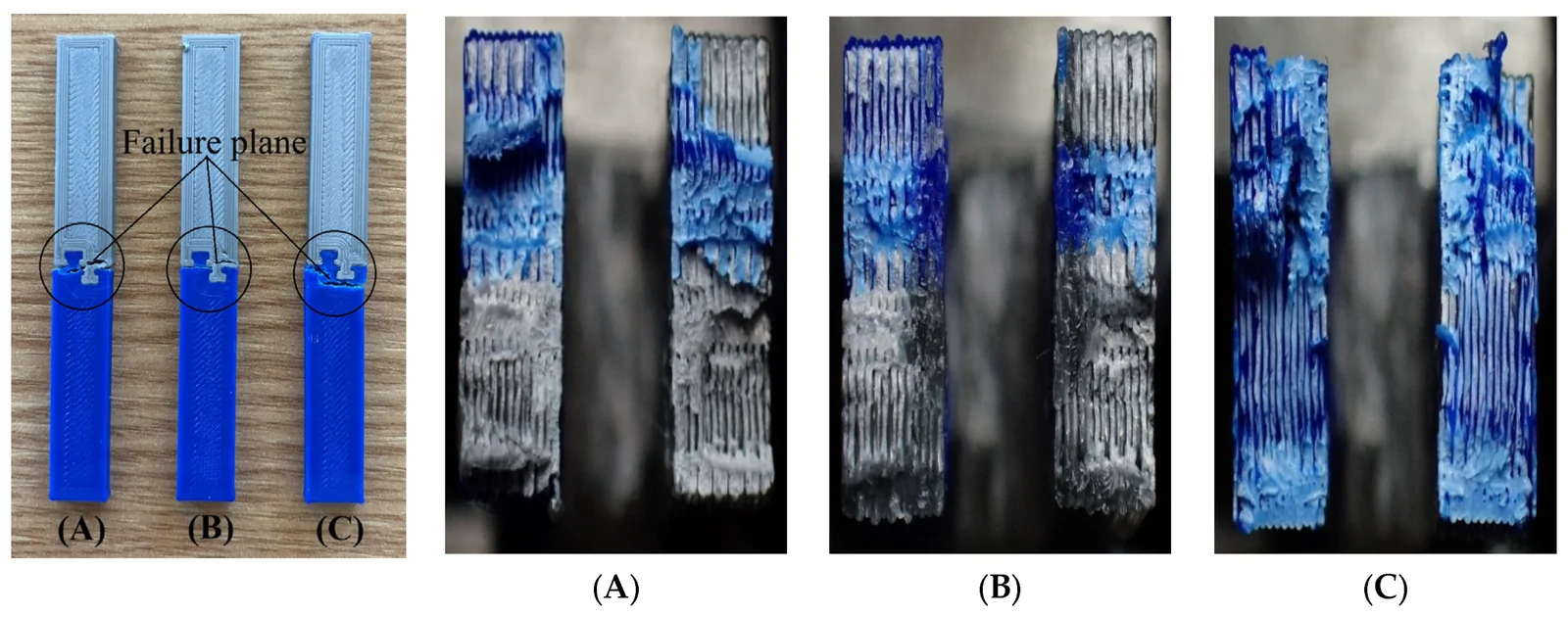
Fracture zones of sample 2, with the 2T interface: (**A**) cross-sectional view of the fracture zone of sample 8.1; (**B**) cross-sectional view of the fracture zone of sample 8.2; (**C**) cross-sectional view of the fracture zone of sample 8.3.

Figure 10 shows the fracture zones for Experiment 8, with the 2T interface. 

**Figure 11:**
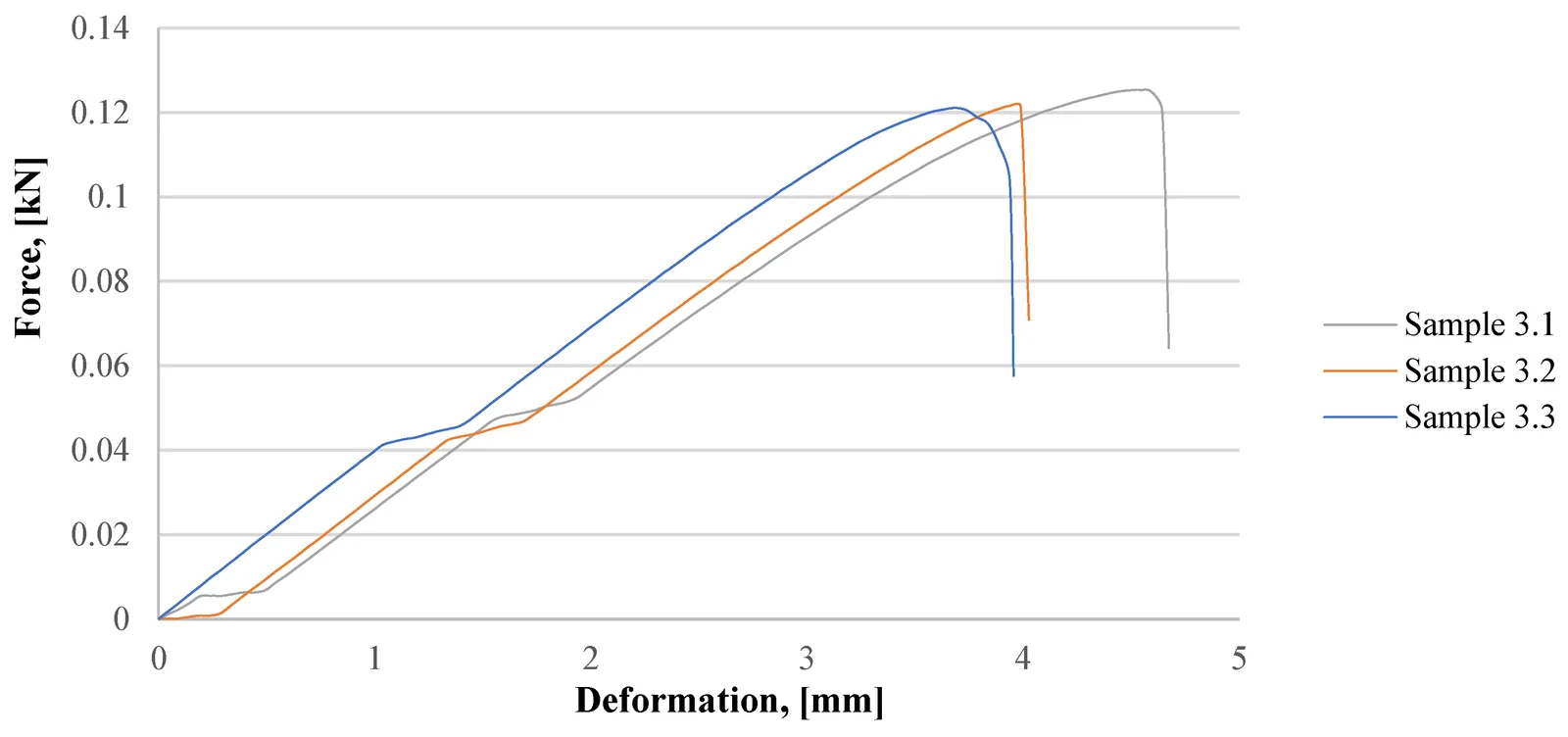
Force–displacement curves for Experiment 3: 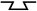 interface.

**Figure 12:**
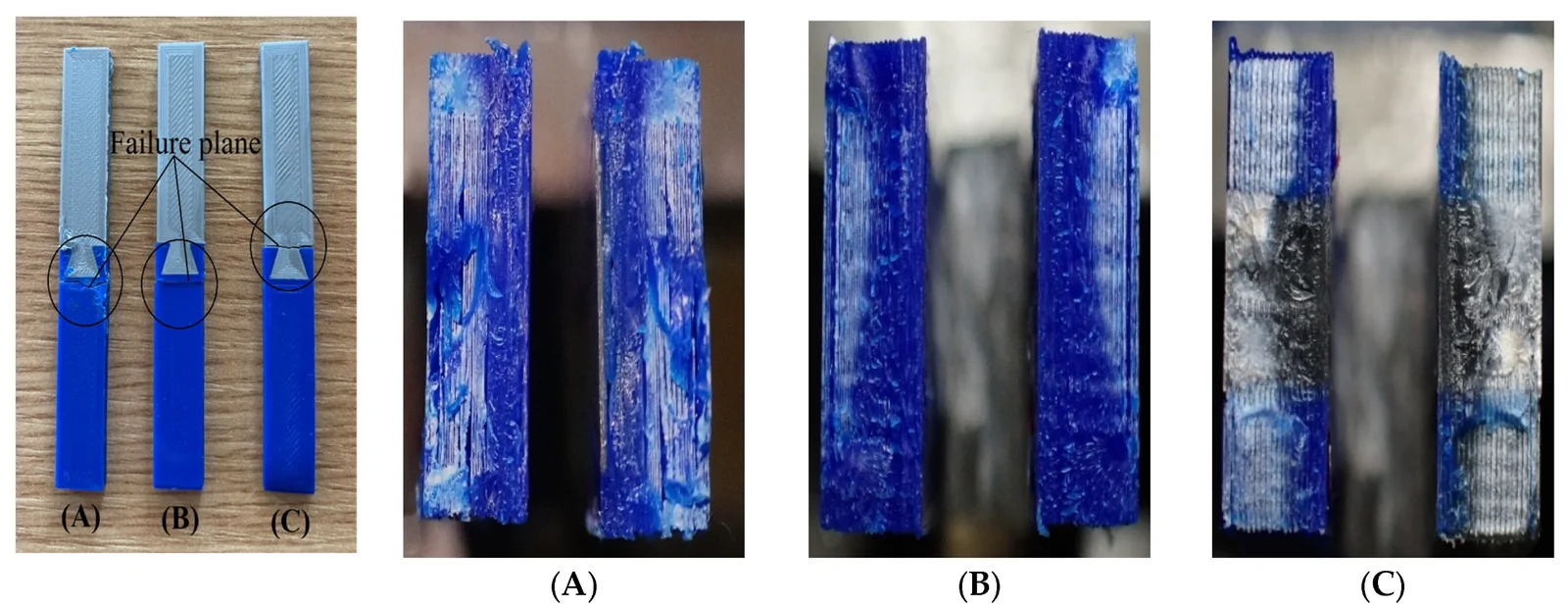
Fracture zones of sample 1, with 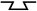 interface: (**A**) cross-sectional view of the fracture zone of sample 3.1; (**B**) cross-sectional view of the fracture zone of sample 3.2; (**C**) cross-sectional view of the fracture zone of sample 3.3.

The force–displacement curves for the two experiments (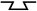 interface and 2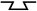 interface) printed using UltraFuse PLA Blue and PLA/PHA Shining Silver materials are shown below (Figure 11 and Figure 12).

Figure 11 shows that sample 3.2 performed best, with a bending strength of 32.14 MPa (Table 8); it withstood the highest load before failure (F = 0.07 kN) and exhibited the greatest deflection (4.03 mm). In conclusion, sample 3.2 has the best mechanical bending strength.

Figure 12 shows the fracture zones for Experiment 3, with a 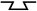 interface.

**Figure 13:**
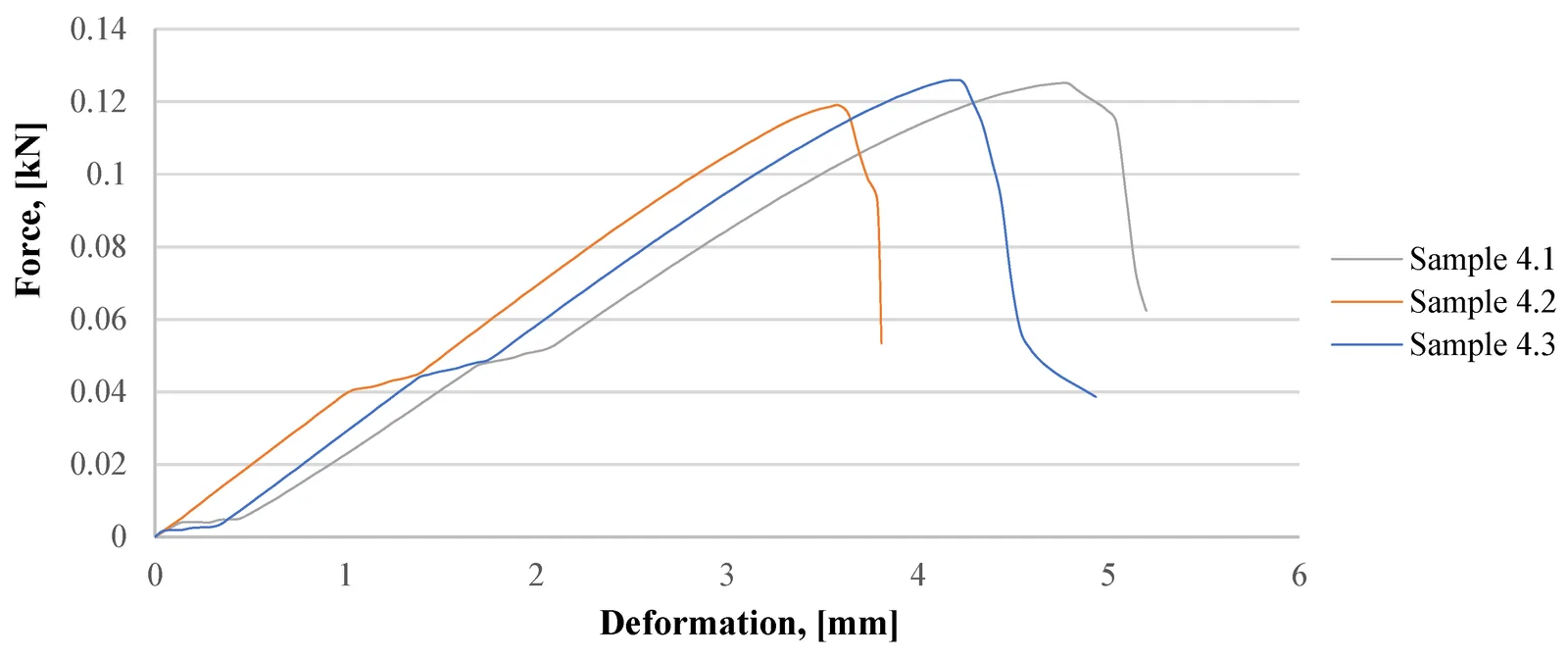
Force–displacement curves for Experiment 4: 2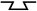 interface.

**Figure 14:**
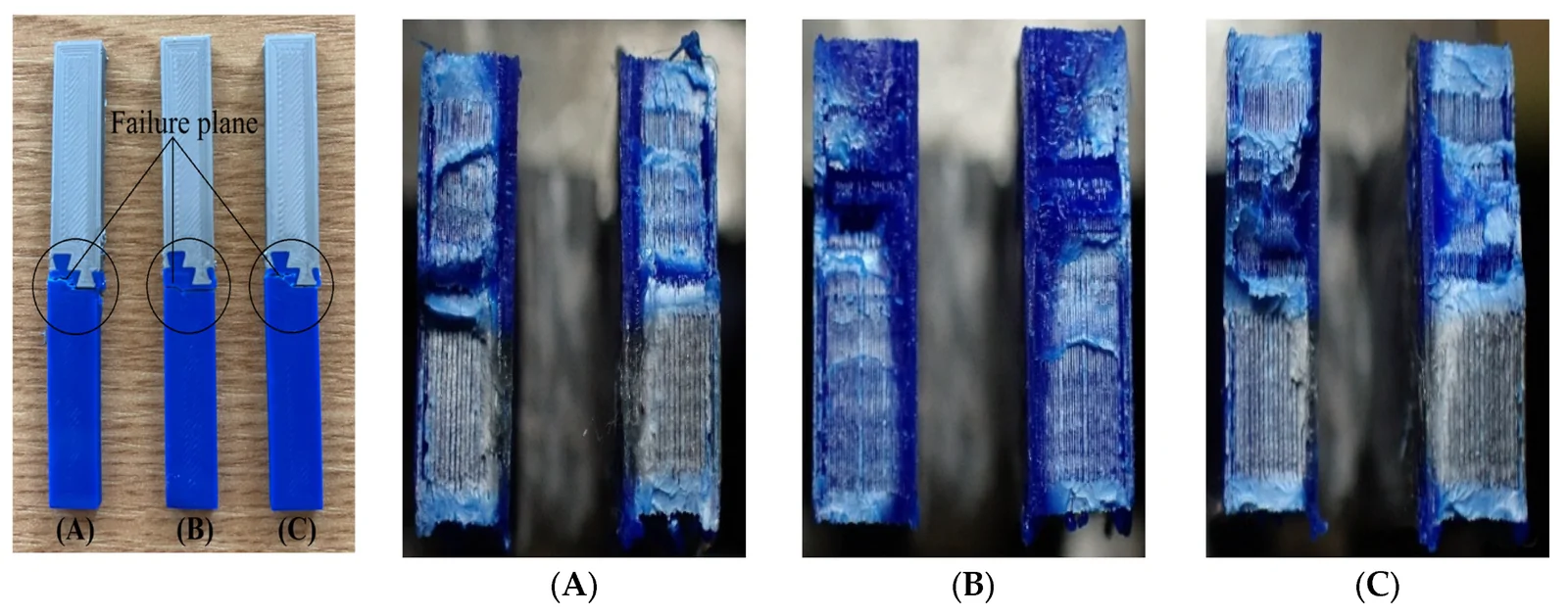
Fracture zones of sample 4, with the 2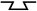 interface: (**A**) cross-sectional view of the fracture zone of sample 4.1; (**B**) cross-sectional view of the fracture zone of sample 4.2; (**C**) cross-sectional view of the fracture zone of sample 4.3.

Figure 13 shows that sample 4.1 performed best, with a bending strength of 28.26 MPa (Table 8); it withstood the highest load before failure (F = 0.06 kN) and exhibited the greatest deflection (5.2 mm). In conclusion, sample 4.1 has the best mechanical bending strength. Figure 14 shows the fracture zones for Experiment 4, with the 2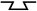 interface.

Table 7 and Table 8 present the results obtained for the experiment, with the best results obtained in the tensile strength test.

### 3.3. Charpy Impact

**Figure 15:**
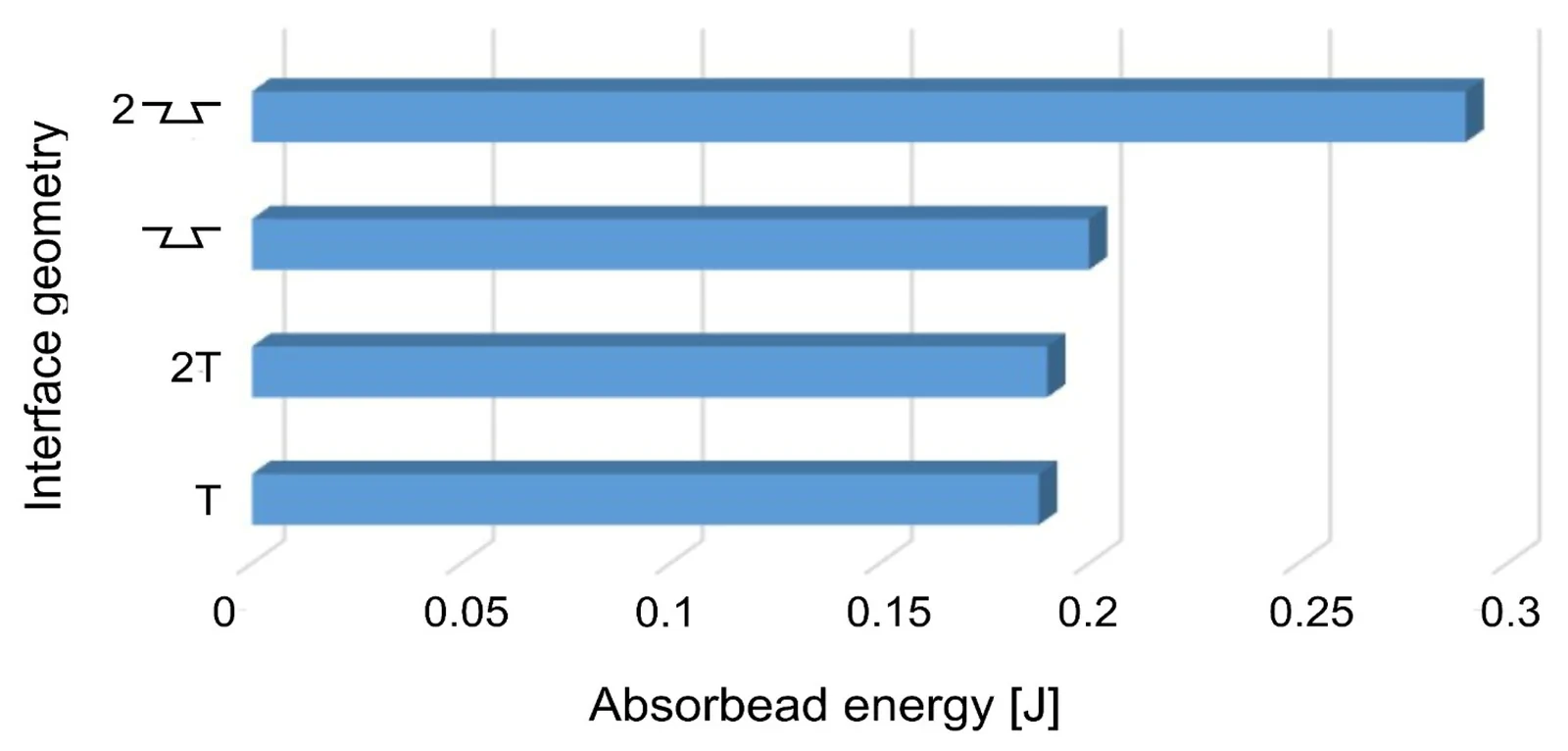
Energy absorbed for all interfaces.

The energy and notch angle values obtained during the Charpy test are presented in Table 9, with the best results obtained during the tensile strength test. The mean absorbed energy (Figure 15) ranges from 0.18 to 0.29 J, values characteristic of brittle–semi-ductile behavior for 3D-printed rigid polymers.

**Figure 16:**
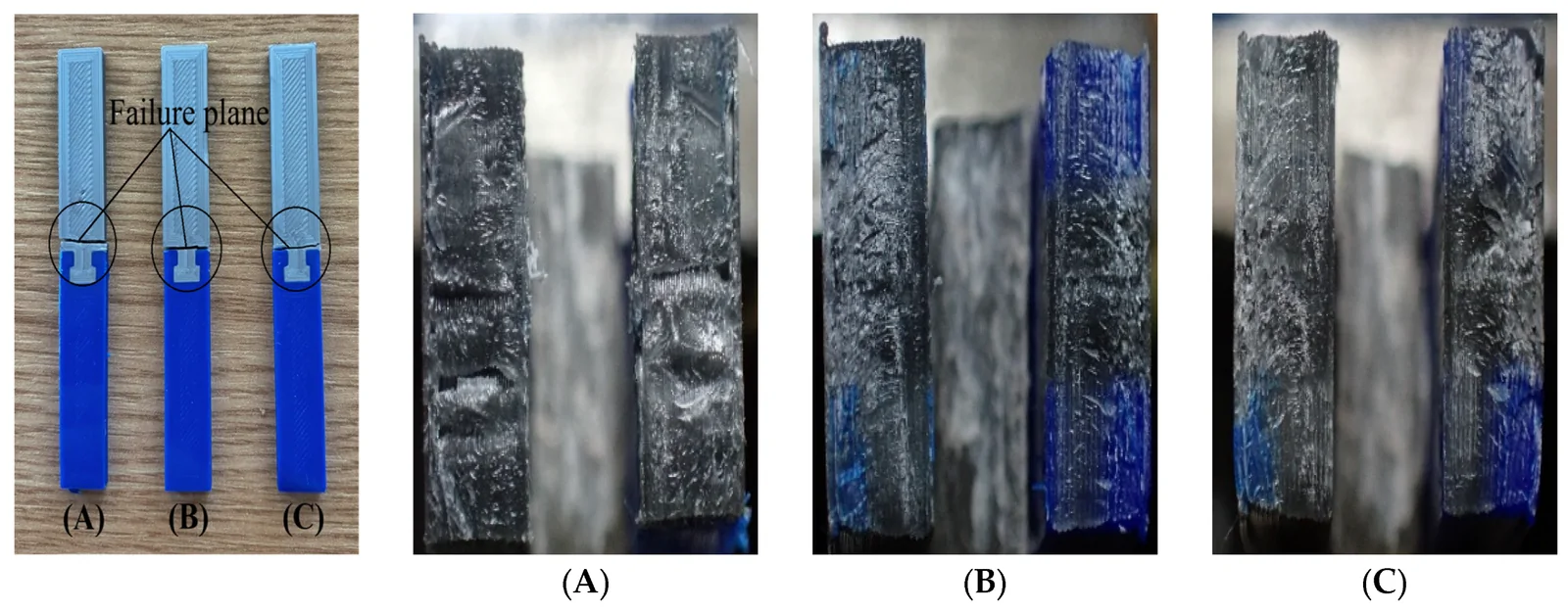
Fracture zones of sample 3, with the T interface: (**A**) cross-sectional view of the fracture zone of sample 3.1; (**B**) cross-sectional view of the fracture zone of sample 3.2; (**C**) cross-sectional view of the fracture zone of sample 3.3.

**Figure 17:**
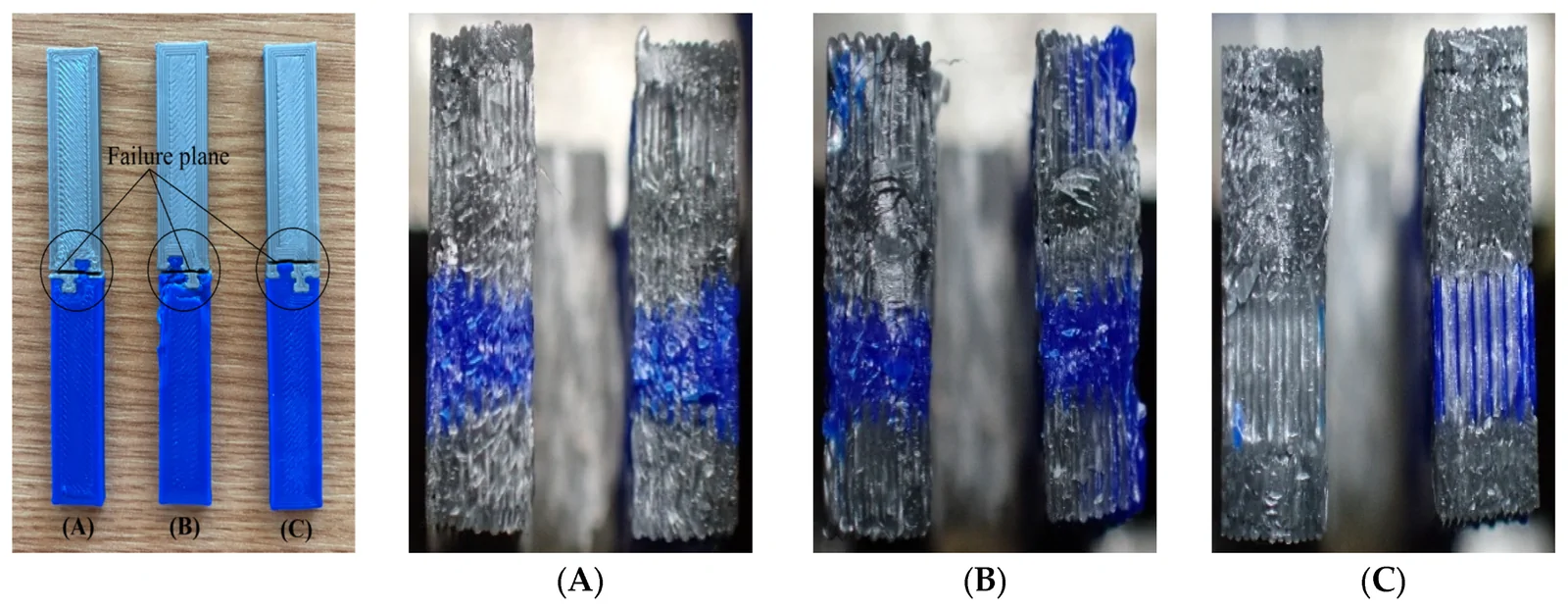
Fracture zones of sample 8, with the 2T interface: (**A**) cross-sectional view of the fracture zone of sample 8.1; (**B**) cross-sectional view of the fracture zone of sample 8.2; (**C**) cross-sectional view of the fracture zone of sample 8.3.

**Figure 18:**
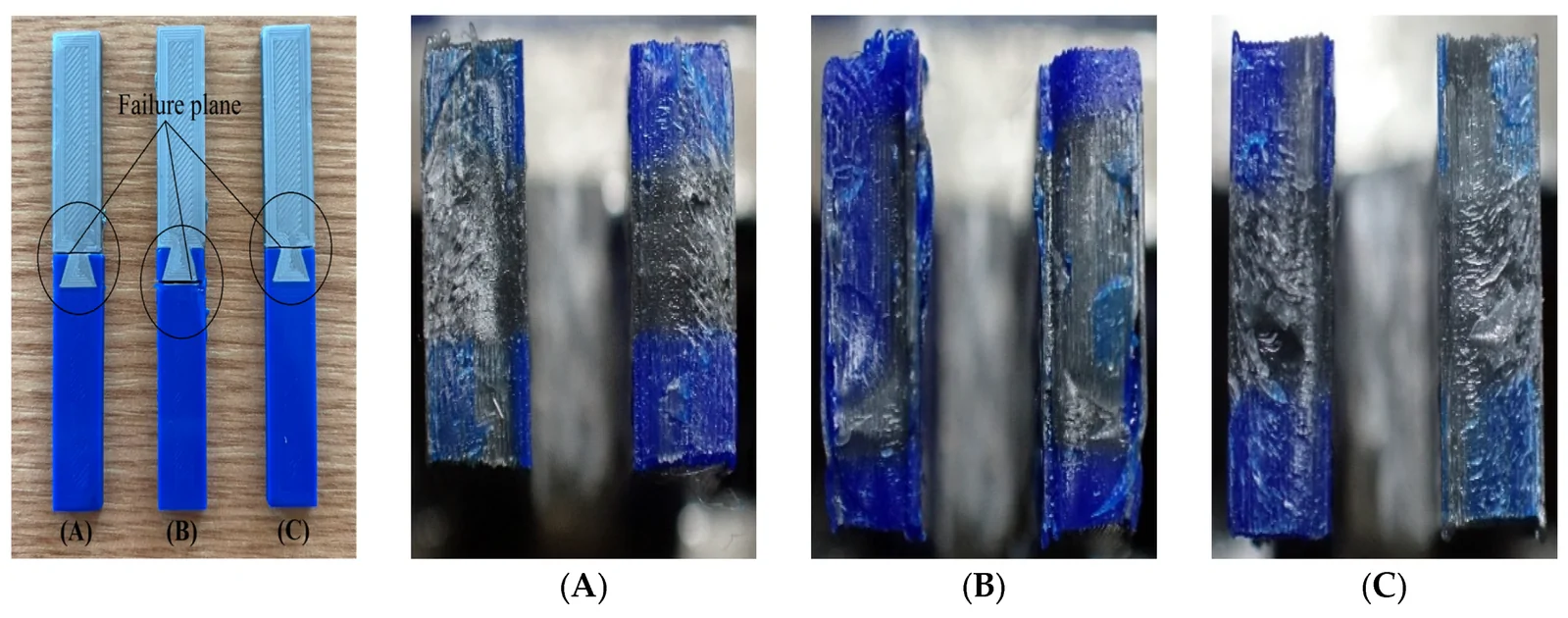
Fracture zones of sample 3, with a 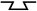 interface: (**A**) cross-sectional view of the fracture zone of sample 3.1; (**B**) cross-sectional view of the fracture zone of sample 3.2; (**C**) cross-sectional view of the fracture zone of sample 3.3.

**Figure 19:**
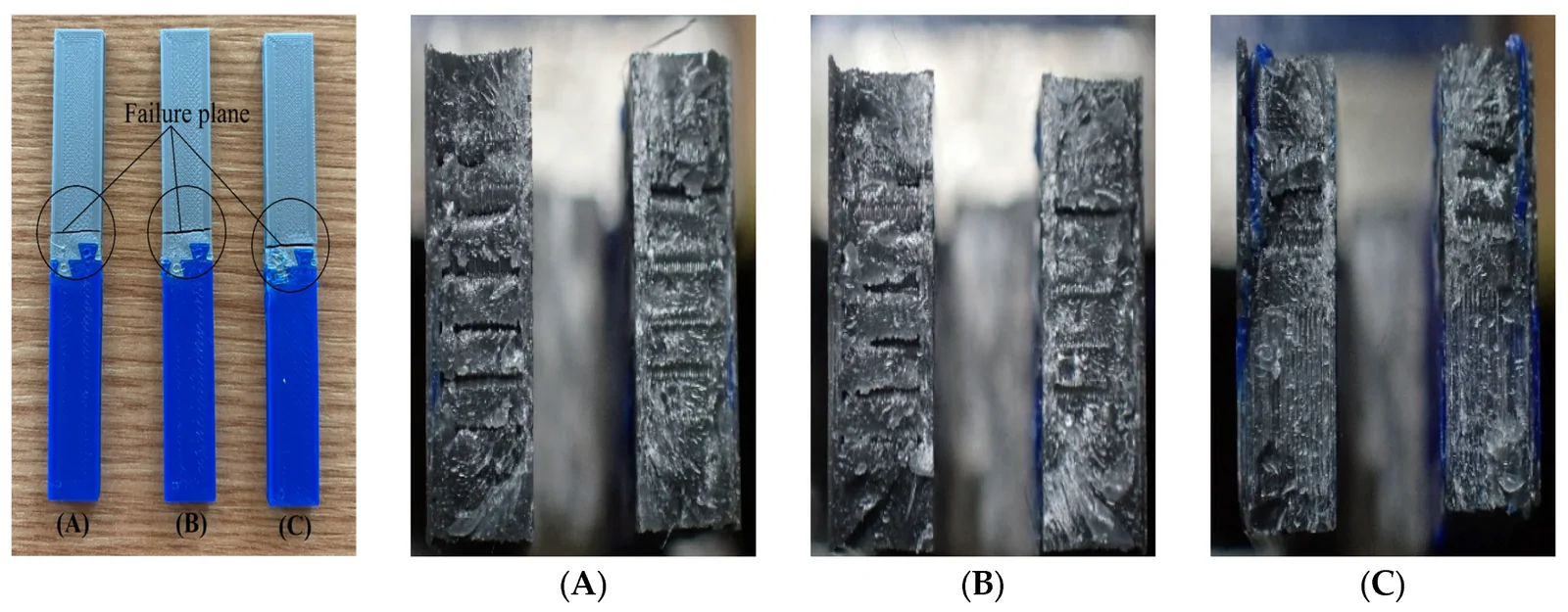
Fracture surfaces of sample 4, with the 2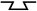 interface: (**A**) cross-sectional view of the fracture surface of sample 4.1; (**B**) cross-sectional view of the fracture surface of sample 4.2; (**C**) cross-sectional view of the fracture surface of sample 4.3.

Next, we will present enlarged images (up to 8× magnification), zoomed in on the fracture surface to observe the structural details of the failure zones for each sample (Figure 16—T interface, Figure 17—2T interface, Figure 18—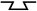 interface, and Figure 19—2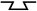 interface).

Images of the fracture surfaces at the T interface (Figure 16) show a predominantly flat fracture zone with a distinct transition between the whitish portions (brittle volumetric fracture) and the interface region. The surfaces appear relatively smooth, with few tear zones, suggesting that the fracture process is dominated by rapid crack initiation and its nearly rectilinear propagation via a cleavage mechanism. The fracture images of the 2T interface (Figure 17) indicate a change in the fracture mechanism: the fracture surfaces become rougher, with areas where the fracture appears to have partially deviated from the normal direction, and the interface appears less like a clear “separation line” and more like a zone integrated into the volume of the specimen. The fact that the 2T interface is printed with a 0.3 mm layer—which is less favorable in terms of interlayer bonding—is partially offset by the mirrored geometry, which increases the effective interaction area and directs the stresses to be distributed across two contact zones. In the images of the 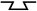 interface (Figure 18), the fracture surfaces exhibit significant irregularities. Extensive areas of tearing and mixed fracture are observed. The images in Figure 19 show fracture surfaces with very significant irregularities.

### 3.4. Topographical Characterization

**Figure 20:**
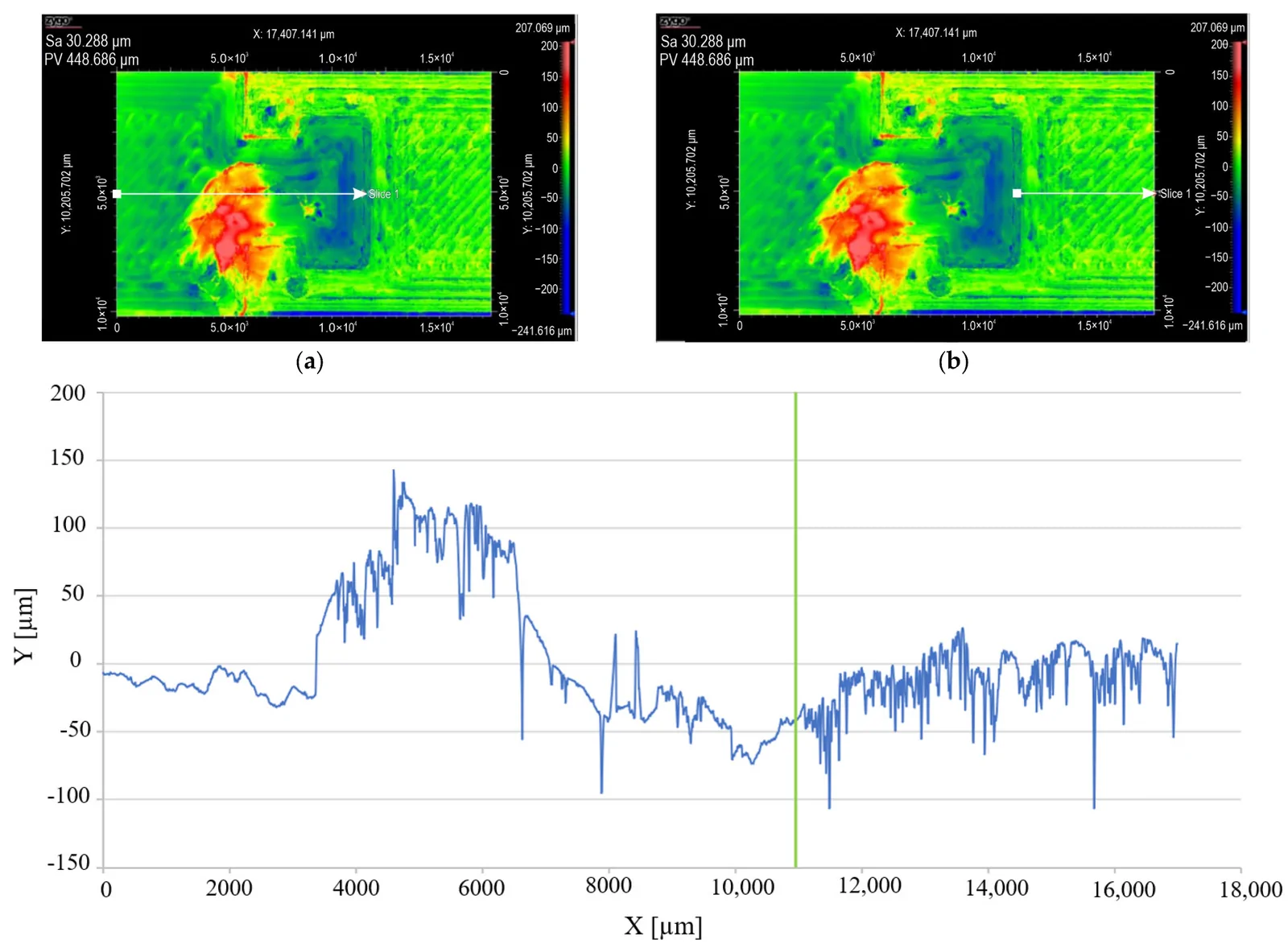
Graphical representation of the variation along slice 1 (Exp. 3, T interface): (**a**) UltraFuse PLA Blue; (**b**) PLA/PHA Shining Silver.

**Figure 21:**
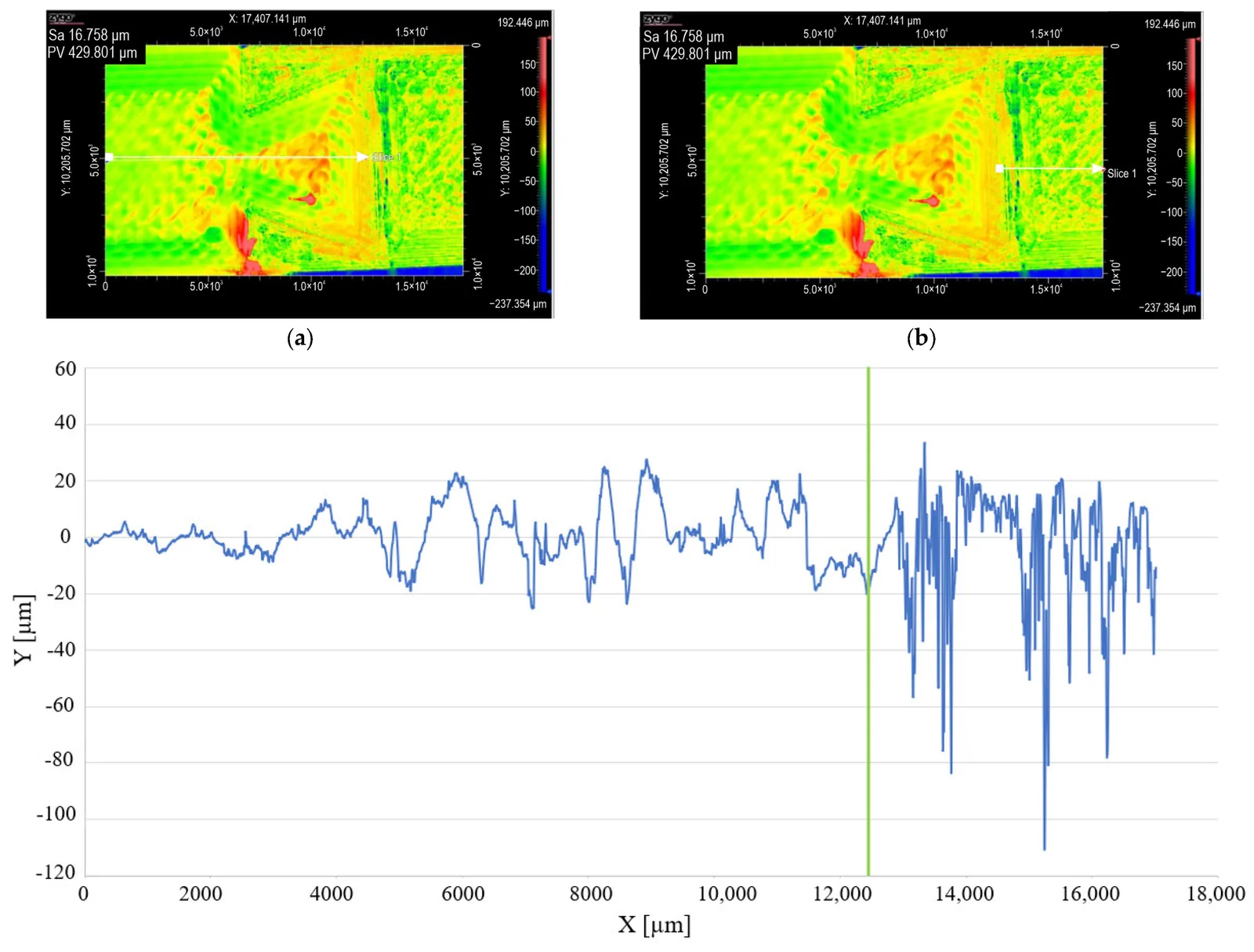
Graphical representation of the variation along slice 1 (Exp. 3, 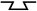 interface): (**a**) UltraFuse PLA Blue; (**b**) PLA/PHA Shining Silver.

To reduce the number of figures, only the longitudinal diagrams for Experiment 3 will be presented below, both for the T-shaped interface (Figure 20) and for the dovetail interface (Figure 21). The analysis was performed from left to right, that is, from UltraFuse PLA Blue toward PLA/PHA Shining Silver.

Figure 20 shows the topographical analysis for the UltraFuse PLA Blue material over the range of (0–11,000) µm, including the interface, and the topographical analysis for the PLA/PHA Shining Silver material over the range of (11.000–17.500) µm. The analysis revealed minor printing defects in the range of (3500–7000) µm, as indicated by peaks with a maximum amplitude of PV = 448.68 µm for Sa = 30.288 µm. Furthermore, the analysis performed on the UltraFuse PLA Blue material revealed, in the range of (7000–11,000) µm, the presence of voids with a maximum depth of approximately 100 µm.

The topographical analysis in the longitudinal direction, for the dovetail interface (Figure 21), was performed over the range of (0–12.500) µm for the UltraFuse PLA Blue material, with no significant peaks or valleys observed. The analysis of the Shining Silver PLA/PHA material was performed over the range of (12.500–17.000) µm, and the peaks and troughs were less than 100 µm.

### 3.5. X-Ray Computer Tomography

XCT reconstructions with porosity analysis were done, showing the top five identified voids and the histogram of the equivalent diameter for each investigated sample. The XCT was performed on each sample individually over a 70 mm length measured from the horizontal axis of the sample, which was secured in a vertical position.

**Figure 22:**
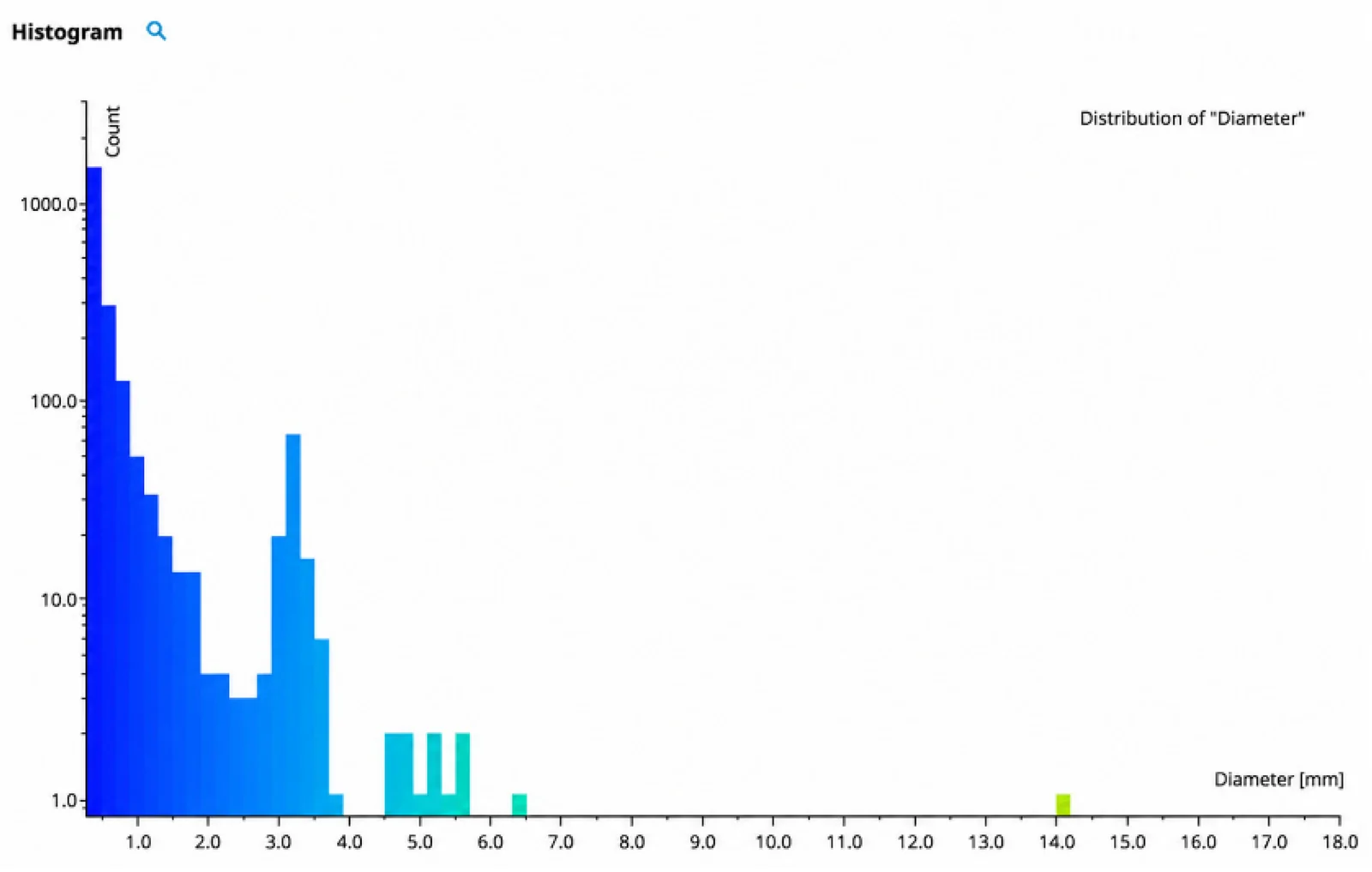
Voids histogram for the T interface.

**Figure 23:**
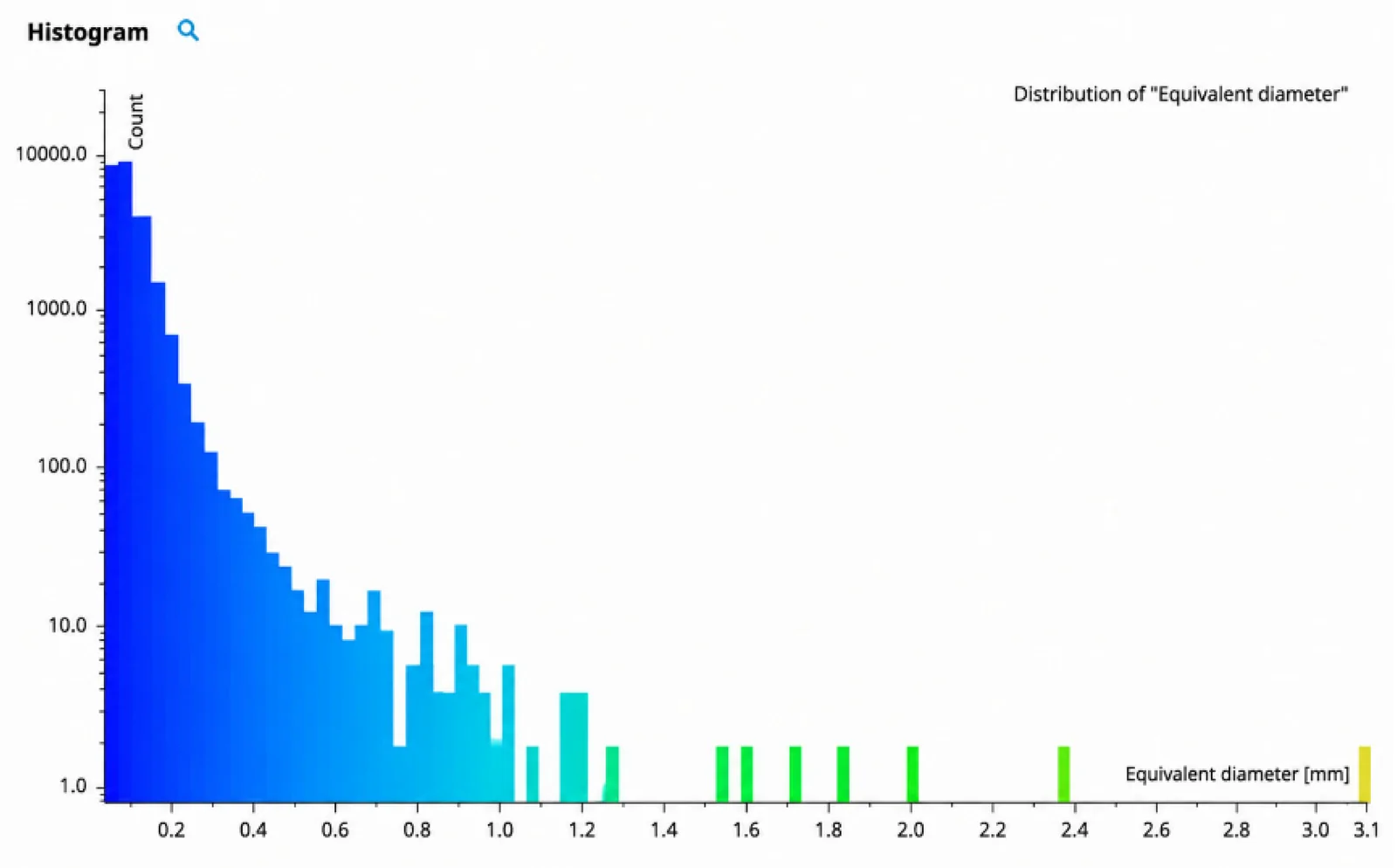
Voids histogram for 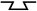 interface.

Thus, in Figure 22, for the T interface (Experiment 3, Sample 3C), the histogram showing the number of voids as a function of the equivalent diameter of the voids is presented, along with an example of a XCT scan with the lowest void percentage of 4.55798% (Table 10) and in Figure 23, for the dovetail interface (sample 3C), the histogram of the number of voids is presented, along with an example of a XCT with the lowest void percentage of 3.63700% (Table 11) obtained for Experiment 3 (sample 3C). Table 10 and Table 11 summarize the data obtained for the T-shaped interface and the 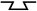 interface.

In the case of the T interface (Figure 1a, Table 10), in Experiment 3, Sample 3B, the highest percentage of voids (5.64214%) was recorded in the lower part of the tested area, i.e., in the UltraFuse PLA Blue material, which explains the fracture in this area of the material. For samples 3A and 3C, the lowest number of voids was recorded in the PLA/PHA Shining Silver material, which explains the occurrence of the break in this material. In Experiment 8, the lowest void percentage was recorded for sample 8B, at 4.65271%, while the highest void percentage was recorded for sample 8A, at 5.90224%.

In the case of the 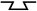 interface (Figure 1b, Table 11), in Experiment 3, the lowest void percentage was recorded for specimen 3C, at 3.637%, which also explains the highest value obtained for σ_max_, at 36.23 MPa. The highest percentage of voids was obtained in sample 3B, at 4.83029%, which also explains the minimum value for σ_max_ of 34.80 MPa and the tensile failure of the PLA/PHA Shinning Silver material.

In the case of samples 3A and 3B, the majority of voids are located in the upper XCT zone, i.e., in the PLA/PHA Shinning Silver material, which explains this material’s failure under tensile stress. In Experiment 4, the lowest void percentage was obtained in sample 4C, at 3.84026%, which also explains the highest σ_max_ value of 34.6 MPa, while the highest void percentage was obtained in sample 4B, at 4.74642%. The highest number of voids was observed in the lower section of the tested samples, specifically in the area containing UltraFuse PLA Blue material, which explains the tensile failure of this material in all three specimens. Furthermore, in the case of sample 4C, the voids were concentrated at the interface, which explains the fracture occurring in the immediate zone of the interface. For sample 4A, the voids are concentrated in the lower section, thus explaining the failure of the UltraFuse PLA Blue material. In the case of sample 4B, the total number of voids, 10.713, is comparable to the total number of voids resulting from the XCT analysis of sample 4C, 8.841, which could explain the failure of the UltraFuse PLA Blue material in the immediate zone of the interface.

## 4. Conclusions

The experimental study employed a design of experiments with three factors and two levels of variation, for a total of eight experiments. For each experiment, three samples were printed using PLA/PHA Shining Silver and UltraFuse PLA Blue materials. The tensile test results showed that the strongest samples were those printed in Experiment 8 for the 2T interface, with a tensile strength of (33.50 ± 0.3) MPa, and those printed in Experiment 3 for the dovetail interface, with a tensile strength of (35.32 ± 0.79) MPa. Furthermore, in Experiment 8 (2T interface), the fracture occurred in the immediate zone of the interfaces in the PLA/PHA Shining Silver material. In the case of the dovetail interface, the results showed the most heterogeneous fractures across the entire experimental setup. For the bending test, the printing parameters corresponding to Experiments 3 and 8 (T interface) and Experiments 3 and 4 (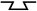 interface) were taken into account. Thus, the bending test results showed that the samples printed using the print parameters corresponding to Experiment 3 (T interface) exhibited the highest bending strength, with a value of 59.75 MPa, and the highest load of 0.13 kN. The mean energy absorbed during the Charpy test ranged from 0.18 to 0.29 J, which explained the brittle–semi-ductile behavior of the materials used.

The topographical analysis was performed in both the longitudinal and transversal directions for the experiments with the best tensile test results. Significant peaks and voids that appeared during printing were identified, most of which were less than 100 µm.

X-ray computer tomography was also performed on the samples that exhibited the highest tensile strength, namely Experiments 3 and 8 for the T interface and Experiments 3 and 4 for the dovetail interface. For these experiments, histograms were plotted showing the number of voids as a function of the equivalent void diameter. XCT demonstrated that the high concentration of voids in the PLA/PHA Shining Silver material (Experiment 3, samples 3A, C) led to the material’s failure under tensile stress, and that the fracture occurred in the immediate zone of the interface, which explained the fracture in this area (Experiment 4, sample 4C).

## Figures and Tables

**Table 1:** Processing conditions for multi-material FFF.

Parameter	Value
Printing speed (mm/s)	60 (in case of outside layer)
Infill, pattern	100% infill, grid
Nozzle diameter (mm)	0.4
Sample orientation	plane
Printing table temperature (°C)	60
Extruder temperature (°C)	215

**Table 2:** The experimental plan.

Exp. No.	Input Parameters		
	Layer Thickness * [mm]	Shell Number **	Interface Geometry ***
1.	−1	−1	−1
2.	−1	−1	+1
3.	−1	+1	−1
4.	−1	+1	+1
5.	+1	−1	−1
6.	+1	−1	+1
7.	+1	+1	−1
8.	+1	+1	+1

* Layer thickness: −1 = 0.1 mm; +1 = 0.3 mm; ** shell number: −1 = 2; +1 = 4; *** interface geometry: −1 = T; +1 = 2T in the mirror, turned around; −1 = 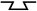; +1 = 2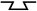 in the mirror, turned around.

**Table 3:** Geometric configurations and dimensions of the investigated interfaces.

T interface	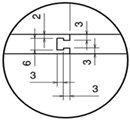	2T interface	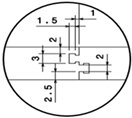
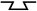 interface	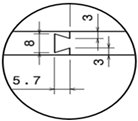	2 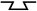 interface	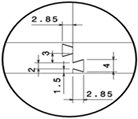

**Table 4:** The best results of tensile properties.

Exp. No.	Layer Thickness [mm]	Shell Number	Interface Type	σ_max_ [MPa]	ε_t_ [%]	E [MPa]
3	0.1	4	T	32.67 ± 0.34	4.28 ± 0.36	1107.12 ± 86.14
8	0.3	4	2T	33.50 ± 0.30	4.26 ± 0.63	1124.71 ± 29.88
3	0.1	4	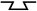	35.32 ± 0.79	4.47 ± 0.35	1173.77 ± 66.02
4	0.1	4	2 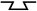	34.36 ± 0.27	4.29 ± 0.03	1163.68 ± 28.98

σ_max_ [MPa]—tensile strength; ε_t_ [%]—tensile strain at break; E [MPa]—Young’s Modulus.

**Table 5:** Tensile strength results for the whole experimental plan (T and 2T interfaces).

Exp. No.	Sample	σ_max_ [MPa]	ε_t_ [%]	σ_y_ [MPa] Offset 0.5%	ε_y_ [%] Offset 0.5%	E [MPa]
1	1.1	28.43	4.76	17.74	2.25	1054.07
	1.2	27.87	4.03	22.77	2.75	1013.07
	1.3	25.30	3.81	22.79	3.15	867.92
Mean		27.20	4.20	21.10	2.72	978.35
SD		1.67	0.50	2.91	0.45	97.81
2	2.1	27.93	4.43	17.26	2.15	1074.14
	2.2	28.20	3.99	21.16	2.45	1094.57
	2.3	28.37	4.3	23.75	2.85	999.16
Mean		28.17	4.24	20.72	2.48	1055.96
SD		0.22	0.23	3.27	0.35	50.24
3	3.1	33.04	4.38	23.41	2.55	1156.04
	3.2	32.36	3.88	25.69	2.75	1157.67
	3.3	32.60	4.59	26.59	3.15	1007.66
Mean		32.67	4.28	25.23	2.82	1107.12
SD		0.34	0.36	1.64	0.31	86.14
4	4.1	31.00	4.21	22.55	2.50	1124.57
	4.2	31.89	4.15	27.24	2.85	1161.78
	4.3	31.48	4.05	26.46	2.75	1176.01
Mean		31.46	4.14	25.42	2.70	1154.12
SD		0.45	0.08	2.51	0.18	26.56
5	5.1	26.31	3.8	21.73	2.80	958.35
	5.2	26.77	3.79	22.38	2.65	1025.97
	5.3	26.71	4.19	21.76	2.75	976.39
Mean		26.60	3.93	21.96	2.73	986.90
SD		0.25	0.23	0.37	0.08	35.01
6	6.1	25.92	4.37	24.12	3.05	938.15
	6.2	25.52	4.7	23.93	3.10	924.48
	6.3	25.48	5.21	21.91	2.75	989.49
Mean		25.64	4.76	23.32	2.97	950.71
SD		0.24	0.42	1.22	0.19	34.28
7	7.1	28.77	3.07	27.26	2.85	1171.53
	7.2	31.23	3.44	27.21	2.80	1187.75
	7.3	30.06	3.66	24.66	2.75	1090.52
Mean		30.02	3.39	26.38	2.80	1149.93
SD		1.23	0.30	1.49	0.05	52.09
8	8.1	33.72	4.94	17.39	2.10	1093.49
	8.2	33.62	4.14	26.56	2.85	1127.61
	8.3	33.15	3.69	29.63	3.05	1153.03
Mean		33.50	4.26	24.53	2.67	1124.71
SD		0.30	0.63	6.37	0.50	29.88

Note: σ_max_ [MPa]—tensile strength; ε_t_ [%]—tensile strain at break; σ_y_ [MPa]—tensile stress at yield-offset 0.5%; ε_y_ [%]—tensile strain at yield-offset 0.5%; E [MPa]—Young’s Modulus; SD—standard deviation.

**Table 6:** Tensile strength results for the whole experimental plan (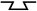 and 2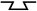 interfaces).

Exp. No.	Sample	σ_max_ [MPa]	ε_t_ [%]	σ_y_ [MPa] Offset 0.5%	ε_y_ [%] Offset 0.5%	E [MPa]
1	1.1	29.50	3.69	24.10	2.60	1139.22
	1.2	28.80	4.00	22.44	2.65	1041.85
	1.3	28.66	3.76	22.54	2.55	1103.41
Mean		28.99	3.82	23.03	2.60	1094.83
SD		0.45	0.16	0.93	0.05	49.25
2	2.1	28.86	4.21	25.27	2.95	1018.00
	2.2	28.07	4.51	19.27	2.35	1053.00
	2.3	28.81	3.91	24.15	2.70	1095.42
Mean		28.58	4.21	22.90	2.67	1055.47
SD		0.44	0.30	3.19	0.30	38.77
3	3.1	34.92	4.14	28.70	2.90	1199.07
	3.2	34.80	4.83	19.42	2.25	1098.84
	3.3	36.23	4.44	28.36	2.80	1223.40
Mean		35.32	4.47	25.49	2.65	1173.77
SD		0.79	0.35	5.26	0.35	66.02
4	4.1	34.07	4.27	26.35	2.70	1184.29
	4.2	34.41	4.27	30.05	3.20	1130.54
	4.3	34.60	4.33	25.50	2.65	1176.21
Mean		34.36	4.29	27.30	2.85	1163.68
SD		0.27	0.03	2.42	0.30	28.98
5	5.1	20.21	2.55	20.17	2.55	983.14
	5.2	18.53	2.53	18.39	2.45	925.94
	5.3	18.50	2.29	17.71	2.26	985.29
Mean		19.08	2.46	18.76	2.42	964.79
SD		0.98	0.14	1.27	0.15	33.66
6	6.1	26.43	4.81	21.22	2.75	944.26
	6.2	27.36	5.48	18.27	2.45	940.55
	6.3	27.47	5.08	22.35	2.80	968.26
Mean		27.09	5.12	20.61	2.67	951.02
SD		0.57	0.34	2.11	0.19	15.04
7	7.1	15.60	1.72	13.72	1.72	1114.19
	7.2	14.54	1.42	10.09	1.42	1085.71
	7.3	15.56	1.71	12.99	1.71	1062.72
Mean		15.23	1.62	12.27	1.62	1087.54
SD		0.60	0.17	1.92	0.17	25.78
8	8.1	23.29	2.35	21.55	2.35	1156.89
	8.2	22.62	2.51	22.11	2.55	1097.75
	8.3	24.59	2.47	21.53	2.45	1088.20
Mean		23.50	2.44	21.73	2.45	1114.28
SD		1.00	0.08	0.33	0.10	37.21

Note: σ_max_ [MPa]—tensile strength; ε_t_ [%]—tensile strain at break; σ_y_ [MPa]—tensile stress at yield-offset 0.5%; ε_y_ [%]—tensile strain at yield-offset 0.5%; E [MPa]—Young’s Modulus; SD—standard deviation.

**Table 7:** Bending results (T and 2T).

Samples with T and 2T Interfaces		Displacement at Break [mm]	Energy at Break [J]	Flexure Strain (Displacement) at Break [%]	Flexure Stress at Break [MPa]	Force at Break [kN]	Time at Break [s]	Flexure Displacement at Maximum Force [mm]	Flexure Strain (Displacement) at Maximum Force) [%]	Flexure Stress at Maximum Force [MPa]	Maximum Force [kN]	Young’s Modulus [MPa]
Exp. 3	3.1	4.23	0.26	3.14	29.53	0.07	84.85	3.89	2.88	54.62	0.12	2305.87
	3.2	4.27	0.21	3.17	6.79	0.01	85.65	3.64	2.70	39.21	0.09	2075.44
	3.3	4.61	0.33	3.42	31.02	0.07	92.32	4.19	3.11	59.75	0.13	2328.20
Mean		4.37	0.27	3.24	22.45	0.05	87.61	3.91	2.90	51.19	0.11	2236.50
SD		0.21	0.06	0.15	13.58	0.03	4.10	0.28	0.21	10.69	0.02	139.93
Exp. 8	8.1	5.81	0.37	4.31	13.85	0.03	116.43	4.34	3.22	51.52	0.11	2038.85
	8.2	4.88	0.30	3.62	20.83	0.05	97.78	4.19	3.11	48.03	0.11	2008.53
	8.3	5.39	0.32	4.00	21.45	0.05	108.00	3.94	2.92	45.31	0.10	2041.10
Mean		5.36	0.33	3.98	18.71	0.04	107.40	4.16	3.08	48.29	0.11	2029.49
SD		0.47	0.04	0.35	4.22	0.01	9.34	0.20	0.15	3.11	0.01	18.19

**Table 8:** Bending results (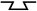 and 2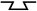).

Samples with 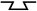 and 2 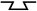 Interfaces		Displacement at Break [mm]	Energy at Break [J]	Flexure Strain (Displacement) at Break [%]	Flexure Stress at Break [MPa]	Force at Break [kN]	Time at Break [s]	Flexure Displacement at Maximum Force [mm]	Flexure Strain (Displacement at Maximum Force) [%]	Flexure Stress at Maximum Force [MPa]	Maximum Force [kN]	Young’s Modulus [MPa]
Exp. 3	3.1	4.67	0.31	3.47	29.08	0.06	93.58	4.54	3.37	56.82	0.13	2177.75
	3.2	4.03	0.25	2.99	32.14	0.07	80.73	3.94	2.92	55.08	0.12	2402.02
	3.3	3.96	0.27	2.93	26.10	0.06	79.34	3.69	2.73	54.86	0.12	2452.57
Mean		4.22	0.28	3.13	29.11	0.06	84.55	4.06	3.01	55.59	0.12	2344.11
SD		0.39	0.03	0.30	3.02	0.01	7.85	0.44	0.33	1.07	0.01	146.28
Exp. 4	4.1	5.20	0.35	3.86	28.26	0.06	104.17	4.74	3.52	56.68	0.13	2107.88
	4.2	3.81	0.25	2.83	24.22	0.05	76.40	3.59	2.66	53.91	0.12	2356.25
	4.3	4.93	0.32	3.66	17.49	0.04	98.92	4.19	3.11	57.09	0.13	2 391.32
Mean		4.65	0.31	3.45	23.32	0.05	93.16	4.17	3.10	55.89	0.13	2285.15
SD		0.74	0.05	0.55	5.44	0.01	14.75	0.58	0.43	1.73	0.01	154.52

**Table 9:** The values for energy and the angle of inclination used during the Charpy test.

Exp. No.	Sample	Interface Geometry	Energy [J]	Pendulum Rebound Angle [°]
3	3.1	T	0.135	104.09
	3.2		0.209	104.22
	3.3		0.221	104.04
Mean			0.18	
SD			0.04	
8	8.1	2T	0.244	103.68
	8.2		0.180	104.67
	8.3		0.151	105.12
Mean			0.19	
SD			0.04	
3	3.1	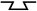	0.197	104.4
	3.2		0.215	104.13
	3.3		0.203	104.31
Mean			0.20	
SD			0.009	
4	4.1	2 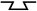	0.268	103.32
	4.2		0.315	102.6
	4.3		0.303	102.78
Mean			0.29	
SD			0.02	

**Table 10:** T interface—centralization of XCT information.

Sample No.	Porosity [%]	Indication Count	Total Indication Volume [mm^3^]	Material Volume [mm^3^]	∑ Voxel	∑ Volume [mm^3^]
3A	5.03301	12,227	80.36584	1516.40869	7,334,623	80.36584
3B	5.64214	21,322	91.70368	1533.63171	8,508,165	91.70368
3C	4.55798	14,739	71.64204	1500.15320	6,618,078	71.64204
8A	5.90224	12,722	92.72211	1478.24170	8,701,816	92.72211
8B	4.65271	12,275	70.27961	1440.23022	6,671,883	70.27961
8C	5.68049	24,107	86.18713	1431.06189	8,622,736	86.18713

**Table 11:** 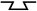
 interface—centralization of XCT information.

Sample No.	Porosity [%]	Indication Count	Total Indication Volume [mm^3^]	Material Volume [mm^3^]	∑ Voxel	∑ Volume [mm^3^]
3A	4.48410	41,292	67.55898	1439.07568	6,127,165	67.55898
3B	4.83029	12,271	75.24384	1482.50488	6,752,803	75.24384
3C	3.63700	15,549	58.32329	1545.28699	5,288,997	58.32329
4A	4.53029	23,015	71.44789	1505.66858	6,457,252	71.44789
4B	4.74642	10,713	75.63515	1517.88599	6,781,273	75.63515
4C	3.84026	8841	57.90508	1449.93762	5,212,911	57.90508

## Data Availability

The raw data supporting the conclusions of this article will be made available by the authors on request.
